# Mesoscale effects of trader learning behaviors in financial markets: A multi-agent reinforcement learning study

**DOI:** 10.1371/journal.pone.0301141

**Published:** 2024-04-01

**Authors:** Johann Lussange, Stefano Vrizzi, Stefano Palminteri, Boris Gutkin

**Affiliations:** 1 Laboratoire des Neurosciences Cognitives, Département des Études Cognitives, INSERM U960, Paris, France; 2 Center for Cognition and Decision Making, Department of Psychology, NU University Higher School of Economics, Moscow, Russia; Università Cattolica del Sacro Cuore Sede di Piacenza e Cremona Facoltà di Economia: Universita Cattolica del Sacro Cuore Facolta di Economia e Giurisprudenza, ITALY

## Abstract

Recent advances in the field of machine learning have yielded novel research perspectives in behavioural economics and financial markets microstructure studies. In this paper we study the impact of individual trader leaning characteristics on markets using a stock market simulator designed with a multi-agent architecture. Each agent, representing an autonomous investor, trades stocks through reinforcement learning, using a centralized double-auction limit order book. This approach allows us to study the impact of individual trader traits on the whole stock market at the mesoscale in a bottom-up approach. We chose to test three trader trait aspects: agent learning rate increases, herding behaviour and random trading. As hypothesized, we find that larger learning rates significantly increase the number of crashes. We also find that herding behaviour undermines market stability, while random trading tends to preserve it.

## 1 Introduction

*Background*: Understanding how markets behave has been one of the central questions in financial market economics. Traditionally, market dynamics are studied as phenomena in themselves with a top-down approach to complexity inference, for example by using statistical or econometric models [1]. Yet trading in real financial markets comes as a result of the collective interactions of human actors, either directly in the form of economic traders, or indirectly in the form of investors imperatives that constrain algorithmic trading strategies [2]. Understanding how these two trading approaches and their gap differ can potentially be bridged using multi-agent systems (MAS), or agent-based models (ABM), which have been sought after by industry practitioners and regulators alike [3]. They are currently an active area of research [4, 5] and tools to study cross-market structure [6], market regulatory impact [2], the law of supply and demand [7], high-frequency trading [8, 9], quantitative easing [10], and other exogenous effects [11]. Modern MAS applied to financial markets have been able to reenact the so-called market *stylised facts* [5], which are statistical properties of the stock price return and volatility signals, observed across different markets, assets and time scales. Stylised facts play an important role in economic theory, because they imply some degree of market memory [12, 13] (e.g. the way historical market prices can help forecasting future prices). Recent trends give MAS research in economics a whole new potential range of realism, coming from the association of two present-day major scientific breakthroughs: i- the steady advances of cognitive neuroscience and neuroeconomics [14, 15], and ii- the progress of machine learning due to the increasing computational power and use of big data methods [16] over the past decade. Even more promising is the synergy of these two fields, with the emergence of machine learning algorithms incorporating decision-theoretic features from neuroeconomics [17, 18], or neuroscience models approached from the angle of machine learning [19, 20].

Studies have also examined cognitive traits and biases in individual financial decision making [14], yet few have revealed the global impact of individual cognitive traits and biases in large populations of economic agents on the quantifiable financial market dynamics [21]. Such approaches have also used methods from reinforcement learning [22–24] or adaptive learning [25, 26]. The framework of reinforcement learning has multiple parallels with decision processes in the brain [17–20]. Reinforcement learning hence computationally offers the possibility to quantitatively study the agent learning side of price formation, which is so crucial to basic market activity. The current literature in financial trading spans over a large and growing number of studies employing reinforcement learning [27]. Their approach ranges from critic-only approach [28–30] to actor-only approach [31–33], to combining actor-critic approach [34] (for a thorough analysis of the three approaches, see [35]). While each approach has its own strengths and weaknesses, and despite studies often starting from unrealistic assumptions [36], RL algorithms have been improved and tested on a number of tasks for trading performance along the last two decades. From discovering predictable structure in foreign exchange markets [33], reinforcement learning algorithms were adapted to portfolio management [37, 38] and trading in the context of individual actor-critic algorithms [39]. RL agents can also be combined together, assigning them different roles (learning when to buy, when to sell or at which price), to solve trading tasks cooperatively [40–43]. RL agents can be designed to compete as traders against each other in the context of self-play RL, and their ability to learn and adapt their own strategy in complex environments offers the opportunity to study how their interaction shapes the evolution of their own strategies. Multi-agent RL (MARL) models [44] can therefore be implemented to simulate bottom-up stock market dynamics and assess emerging order book dynamics from limit orders issued by heterogeneous agents [45]. However, the recent reinforcement learning approaches have been calibrated by financial data that is incomplete at best [22–24], or have not been built over a full system of autonomous reinforcement learning agents to emulate the market [22, 46]. Even so, such studies confirm a trend of recent interest in reinforcement learning applied to financial MAS [28, 46–48] or order book models [49–51].

*Our approach*: We base our work on our reinforcement learning MAS stock market simulator—SYMBA (*SYstème Multi-agents Boursier Artificiel*). IN SYMBA all of the agents are autonomous and are endowed with reinforcement learning [52], by which they forecast stock prices and send individual transaction orders to a centralised order book. In a previous publication [53], we detailed the cautious calibration procedure of SYMBA to real stock market data, also see Supplementary Material. In [54], we then studied how its agents learn and acquire new trading strategies over time. SYMBA hence emulates the microstructure of a financial stock market through a bottom-up approach to system complexity, via these autonomous economic agents (e.g. investors, institutions) and their economic transactions (e.g. buying, selling, holding stocks). Each individual agent is modelled according to two distinct features: i- a reinforcement learning algorithm to develop its own skills for price forecasting and stock trading (i.e. each agent learns to forecast and trade over time), ii- such an agent learning process framed for a rather chartist or fundamentalist approach to stock price valuation. At each time step of the simulation, the agents individually learn to either buy, sell or hold stocks, in a given number and at a given price. To do this, each sends its transaction order to an order book that is common to all agents and which sorts and matches the transaction orders received. By reinforcement learning, the agents analyze the results (rewards) of their past investments (actions) based on the actions they chose during specific market phases (states). As market prices unfold over time, agents learn to adapt and refine their strategies (policy). The law of supply and demand, and other key phenomena to price formation [55, 56] such as illiquidity and bid-ask spread formation are thus reenacted. We then compare the simulated price and volume time series of each stock to real financial data. The SYMBA MAS parameters can be calibrated so that the output matches real stock markets, thereby measuring the collective role played by the learning parameters of the agents in a quantitative way. Each agent learns to forecast and trade autonomously by reinforcement learning with a long-only equity strategy, and each agent’s discretionary asset pricing process relies on learning to weight both chartist and fundamentalist inputs. These features allow us to plumb the financial market from the macro- to the microscopic level: in [53], we compared the overall stock market return statistics to real world data, while in [54] we characterised the trading performance of the agents from the learning dynamics of their trading strategies over time.

*This study*: In this study we use our stock market simulator to investigate the mesoscopic effect of the agent learning parameters on the emergent collective market dynamics. More specifically, we chose to test the effects of three manipulations in traders behaviour on stock market stability: learning rate amplification (Section 4), herding behaviour (Section 5) and noise trading (Section 6). In the first manipulation, increased learning rates will make agent more sensitive to more recent outcomes, reflecting the more recent states of market microstructure. The agent will therefore tend to select actions that were successful most recently. Secondly, we study the impact of herding behaviour, which we view as a source of market reflexivity (i.e. market endogeneity) [57] that can lower market stability. Herding can arise from imitating the trading or following the investment advice from another agent (e.g. renowned investor, trader, or analyst, etc.) in the form of recommendations or reviews. However, herding behaviour is more than imitation, it may also include common responses to aligned expectations (e.g. when the price of a given stock starts decreasing) or heuristics (e.g. agents stopping to buy a stock because of an influential investor starting to sell it, etc.). Finally, we study how information asymmetry among agents influences market stability. With the ever-increasing amount of big data, information has an ever increasing impact on price formation and markets. We examine the role played by agent information on market price formation [58], by introducing an increasing percentage of “noise traders”, i. e. agents trading randomly [59].

*Motivation for trait selection*: There are many individual traits that are studied in behavioral economics, and that could be probed via the agent reinforcement learning framework in our model. However, in this paper we limit ourselves to study the impact of the traits of a collective of agents on the market *at the mesoscale*. As we show below, the exploration of learning rate variations, herding behavior, and the role of noise traders allows for a multifaceted analysis of market dynamics, ranging from extreme volatility to more stable conditions. This selection of manipulations and agent traits allows one to study the effects of both agent rationality (e.g. noise trading) and collective actions (learning rate and herding). The learning rate in reinforcement learning agents is a critical factor in determining the adaptability of agents to new market information. Faster learning rates result in agents that heavily weigh recent experiences, leading to quick alterations in trading strategies. This can significantly influence market trends and volatility. For example, [60] explored how varying learning rates [35] among traders may lead to market instabilities, and conversely, slower learning rates contribute to more steady but potentially less agile trading strategies. These insights underscore the importance of understanding how the speed of learning among market participants can shape overall market behavior. The tendency of investors to mimic the actions of a larger group (e.g. herding) can cause significant market disruptions, potentially leading to overreactions or the formation of price bubbles. Our study on herding behavior, inspired by the findings of [61], examines how collective actions, driven by herding, can critically influence market quality, offering insights into the mechanisms behind correlated trading behaviors. Finally, exploring the impact of noise traders (i.e. agents who act randomly and without coherent strategies) is crucial for a comprehensive understanding of market dynamics in that it deals with agent rationality. As demonstrated by [62], noise traders can introduce an element of unpredictability that counters the effects of more strategic traders, affecting market liquidity and overall stability. By incorporating these agents, one can study the intricate interplay between systematic strategies and trading randomness, as well as the potential for irrational behavior to impact market conditions.

*Working hypotheses*: We hypothesise that each one of these manipulations will have a unique impact on the emergent structure of the market: (1) faster learning in agents will lead to increased agent bankruptcies and more frequent crashes, (2) herding behavior among the agents should lead to market instabilities and (3) an increasing number of noise traders brings financial stability to the whole market. Noise traders should provide more liquidity (and higher trading volumes in both bid and offer), as they keep a balance between bid and ask orders, hindering trends towards either bear or bull markets. These experiments are of particular interest because all the three manipulations and their impact on market stability relate closely to a core concept in finance: market memory [63, 64]. This topic has sparked an important historical debate in the scope of the well-known efficient-market hypothesis [65, 66]), which states that in a market large enough where information spreads instantaneously to all agents, these react rationally and immediately to it, so that market prices are always at their fair value, and no consistent profit can be consistently earned over time by investors buying undervalued stocks and selling overvalued ones, or exploiting historical data patterns so as to forecast future data.

*Structure*: This paper is organized as follows. A short primer on reinforcement learning is first given in Section 2 (with more details in the Supplementary Material section). Then, Section 3 describes our model’s general architecture (with its iteration procedure), its agents (with their reinforcement learning algorithms to forecast and trade stocks), and its order book (with its double auction limit orders procedure). For the sake of clarity, the entire code of SYMBA is available to the community on GitHub [67]. We also provide as Supplementary Material a detailed sum up of the main results of the calibration procedure from [53] to validate the model. In Section 4, we study the impact of the reinforcement learning rate on agent performance and overall market dynamics. In Section 5, we study the impact of agent herding and market reflexivity (i.e. market endogeneity). Finally, in Section 6, we study via different populations of noise traders how information asymmetry among agents influences the market stability. ‘In the supplementary Material we lay out in more detail how the model was calibrated and other technical issues.

## 2 Reinforcement learning

We here briefly review the basics of reinforcement learning theory that pertain to this study. Together with supervised and unsupervised learning, reinforcement learning has been termed one of the three paradigm shifts of machine learning [68], and is today at the forefront of almost all breakthroughs in machine learning research. Like many other machine learning methods, reinforcement learning has its roots in behavioural psychology and decision theory [69]. In reinforcement learning, we consider an agent in a given *state* s∈S of its environment that must learn the best way to consistently receive a preset *reward* r∈R from this environment through a selection of its possible *actions* a∈A. The states and actions can be defined as more or less complex concepts, and notice the rewards can be positive or negative. In the beginning of the task, the agent is completely agnostic as to which actions are best to use: it will have to learn this on its own by trial and error, through a formalism known as the exploration vs. exploitation procedure. The goal of the agent is to find its policy *π*(*s*, *a*) = *Pr*(*a*|*s*), which is the set of probabilities associated to each of the agent’s action selection within each possible state of its environment, so as to maximise its (delayed) rewards in a dynamic (and often stochastic) environment. A more expanded review of reinforcement learning is presented in the Supplementary Materials.

## 3 Model

*Features*: We have previously showed SYMBA performance and its calibration process to real stock market data [53] and see Supplemenatry Materials. The SYMBA agents utilize reinforcement learning to interact with the market through the order book. Each agent’s unique asset pricing process, which balances chartist and fundamentalist perspectives, allows for an analysis of the impact of various learning parameters on the market at the mesoscale (i.e. between micro- and macroscale). This is achieved by comparing our simulated data with real stock market data. The data we use for such a comparison comes from a data-feed of 642 stocks from the London Stock Exchange that have been continuously traded over the years 2007 to 2018 (these thus comprise a survivorship bias). Our data consists of professional-grade, end-of-day stock market quotes. These quotes include the date, opening price, highest price, lowest price, closing price, and trading volume for each day. Importantly, these figures are directly sourced from the London Stock Exchange (LSE) and are not a compilation of data from smaller exchanges (i.e. consolidated data). Additionally, our dataset includes precise information on stock splits. This ensures that our analysis is not mistakenly influenced by extreme market events that are actually just the result of stock splits.

*Architecture*: SYMBA (see pseudo-code in the Supplementary Material section, on Fig 11) is structured along two major parameters: i- a number *I* of agents aiming to learn actions that maximise over time the net asset value of their individual portfolio, which consists in risk-free assets (bonds) and a number *J* of stocks (equity), and ii- a number *J* of different double-auction limit order books, each compiling the transactions of a stock *j* ∈ *J* at each time step *t* of the simulation. For the results shown in Section 4 to 6, we consider the case *J* = 1, and instead use batches of S∈R simulation runs for the statistical validation of these results. Each agent is initialized in such a way that it is agnostic wrt. both price forecasting and trading. An agent autonomously learns these by two distinct reinforcement learning algorithms, and sends (or not) at each time step *t* a transaction order to the order book for a specific number of each stock *j* to buy or sell. Otherwise the agent simply holds its position and waits for a better time to trade. At each time step, each order book thus collects the transaction orders of all agents and processes them by sorting the bid orders in a descending way, and the ask orders in an ascending way. The order book then matches the orders for transactions at mid-price at each level, starting from the top, until bids no longer exceed offers. That latest effective transaction at the lowest possible level then defines the market price of stock *j* at next time step *P*^*j*^(*t* + 1). The total number of stocks transacted at time step *t* is its traded volume at next time step *V*^*j*^(*t* + 1). The absolute difference between the average of all bids and asks is its spread *W*^*j*^(*t* + 1). The market price at time *t* = 0 is set by default at *P*(*t* = 0) = *£*100.

*Agents*: For their own asset pricing, agents approximate by cointegration [70] another time series $T^{j}\left(t\right)$ generated at time *t* = 0, which corresponds to the fundamental prices of asset *j* (as in other models [71, 72]). The inspiration for the profile of $T^{j}\left(t\right)$ is the *enterprise value* [73], which gives the theoretical price at which the company issuing the stocks would be acquired. The enterprise value can give a rough fundamental stock price estimate, if divided by the total number of stocks outstanding. For more details on the way $T^{j}\left(t\right)$ is generated and then individually approximated by each agent, we refer the reader to [53]. The weight given to this fundamentalist valuation or to the market price is learned by each agent, so that agents range from chartists to fundamentalists (see below). Agents trade as such for a learning phase of 1000 time steps, after which all their portfolio assets are reset to their initial values, and the simulation then let to run for statistical inference and microstructure study. Past this learning phase, we consider a simulation of *T* time steps, where one time step typically represents a trading day, and thus *T*_*w*_ = 5, *T*_*m*_ = 21, *T*_*y*_ = 286 correspond to a trading week, month, and year on the London Stock Exchange, respectively. The entire code in C++ of SYMBA is available on GitHub [67].

**Fig 1:**
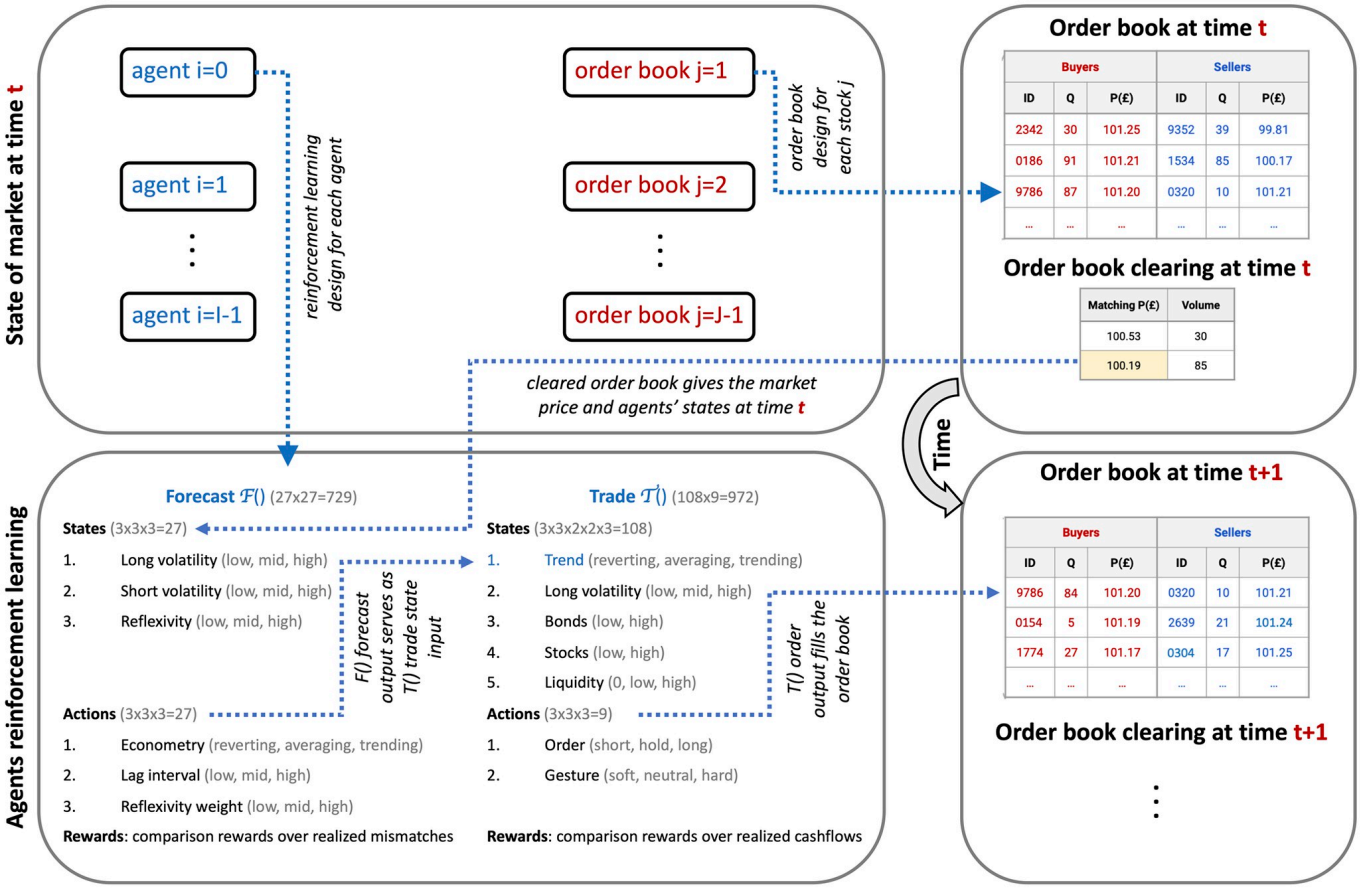
Schematic of the SYMBA stock market simulator and its operational dynamics. This figure presents an integrated view of the SYMBA simulator, emphasizing the dual-level interaction within the simulated financial market. At the core of the system, individual agents (bottom-left) utilize two distinct reinforcement learning algorithms, $F^{i}$ for forecasting and $T^{i}$ for trading, to independently formulate and execute trading strategies at each simulation step. These strategies are then aggregated at the market level through a centralized double-auction order book (top-right). The order book directs market dynamics by matching buy and sell orders from different agents, effectively determining market prices and volumes (bottom-right). This figure illustrates the iterative loop of agent decision-making and market adjustment (top-left), which collectively shapes the emergent macroscopic market behavior. By simulating the interplay between individual agent strategies and market-level effects, SYMBA provides insights into how individual behaviors and collective market responses yield in a complex financial ecosystem.

Each agent is initialized with distinct parameters. For every stock *j* at each time step *t*, an agent runs a reinforcement learning algorithm, $F^{i}$, for price forecasting, followed by another algorithm, $T^{i}$, to determine trading strategies based on $F^{i}$’s outcomes. Fig 1 shows a general overview of the iteration procedure of the simulator.

i- *Agent parameters*: Each agent is initialised at time *t* = 0 with a specific set of seven parameters, as well as two parameters setting their initial portfolio, as detailed in [53]. Let U(),U{},N(),N{} respectively denote the continuous and discrete uniform distributions, and the continuous and discrete normal distributions. The agent parameters include:

- Risk-free assets of value $A_{\text{bonds}}^{i}\left(t=0\right)∼N\left(0,10^{4}\right)$ and a number of stocks *Q*^*i*, *j*^(*t* = 0) drawn from a discrete positive half-normal distribution $N^{+}\left\{0,100\right\}$, amounting to a value of its stock holdings $A_{\text{equity}}^{i}\left(t=0\right)=\sum_{j=0}^{J}Q^{i,j}\left(t=0\right)P^{j}\left(t=0\right)$.
- An investment horizon $\tau^{i}∼U\left\{T_{w},6T_{m}\right\}$ corresponding to the number of time steps after which the agent liquidates its position.
- A memory interval $h^{i}∼U\left\{T_{w},T\right\}$ corresponding to the size of the rolling time interval used by the agent for its learning process.
- A transaction gesture $g^{i}∼U\left(0.2,0.8\right)$ scaling with the spread, and related to how far above or below the value of its own stock pricing the agent is willing to trade and deal a transaction.
- A reflexivity amplitude parameter $\rho^{i}∼U\left(0,100\%\right)$ gauging the weight given by the agent to fundamental or chartist valuation of the stock.

Two agent parameters are particularly relevant to the present study:

- The learning rate $\beta_{i}∼U\left(0.05,0.20\right)$ of both RL algorithms $F^{i}$ and $T^{i}$ (see below): its role is to scale the update of the state-action probabilities when learning, for any action *a*^⋆^ deemed optimal in state *s* at time *t*, by increasing the policy probability associated with this action compared to the other actions, ∀*a* ≠ *a*^⋆^:
  $$\begin{array}{c} \pi_{t+1}\left(s,a^{⋆}\right)=\pi_{t}\left(s,a^{⋆}\right)+\beta \left[1-\pi_{t}\left(s,a^{⋆}\right)\right] \end{array}$$ (1)
  $$\begin{array}{c} \pi_{t+1}\left(s,a\right)=\pi_{t}\left(s,a\right)+\beta \left[0-\pi_{t}\left(s,a\right)\right] \end{array}$$ (2)
  Its boundaries are drawn from the literature in neuroscience on the values of the learning rate [17, 18, 74].
- The drawdown limit $l_{i}∼U\left(50\%,60\%\right)$: this is the threshold of the year-to-date peak-to-bottom loss in net asset value, beyond which the agent is listed as bankrupt and unable to interact with the market anymore.

ii- *Agent forecasting*: The states of the forecasting algorithm $F^{i}$ are described by: a longer-term price volatility $s_{0}^{F}$ (equal to 0 for low, 1 for mid, 2 for high), a shorter-term price volatility $s_{1}^{F}$ (0 for low, 1 for mid, 2 for high), and the gap between its own present fundamental valuation and the present market price $s_{2}^{F}$ (0 for low, 1 for mid, 2 for high).

Out of these states, the agent chooses an action in order to optimise the price prediction at its investment horizon *τ*^*i*^: the type of econometric forecast $a_{0}^{F}$ (equal to 0 for mean-reverting, 1 for averaging, 2 for trend-following), the size of the historical lag interval for this econometric forecast $a_{1}^{F}$ (equal to 0 for short, 1 for mid, 2 for large), and the weight given to its reflexivity amplitude parameter *ρ*^*i*^ for price estimation $a_{2}^{F}$ (equal to 0 for low, 1 for mid, 2 for large).

Then, the reinforcement learning algorithm $F^{i}$ computes the percentage difference between the agent’s former stock price prediction *H*^*i*,*j*^(*t* − *τ*^*i*^) performed *τ*^*i*^ time steps ago, and its present realisation *P*^*j*^(*t*): |*H*^*i*,*j*^(*t* − *τ*^*i*^) − *P*^*j*^(*t*)|/*P*^*j*^(*t*). This difference is recorded it at each time step in a time series that is continually sorted in ascending order and truncated to keep a size corresponding to the agent memory interval *h*^*i*^. The associated percentile corresponding to this value at time step *t* sets a discrete value of returns $r^{F}$ in the set {4, 2, 1, −1, −2, −4} if it respectively belongs to the intervals [0%, 5%(, [5%, 25%(, [25%, 50%(, [50%, 75%(, [75%, 95%(, [95%, 100%]. Hence, the rewards of these performed actions are defined via the mismatches between past forecasts at time *t* − *τ*^*i*^ and their eventual price realisation at time *t*. These feed a direct policy update with new action probabilities for the agent in such a state.

iii- *Agent trading*: The states of the trading algorithm $T^{i}$ are as follows: (i) the trend of the price forecast of the previous algorithm $s_{0}^{T}$ (equal to 0 for decreasing, 1 for stable, 2 for increasing), (ii) the price volatility $s_{1}^{T}$ (equal to 0 for low, 1 for mid, 2 for high), (iii) the level of the agent risk-free assets compared to its initial values $s_{2}^{T}$ (equal to 0 for low, 1 for high), (iv) the level of the agent stock holdings compared to its initial values $s_{3}^{T}$ (equal to 0 for low, 1 for high), and (v) the stock liquidity based on previous exchanged volumes $s_{4}^{T}$ (equal to 0 for zero, 1 for low, 2 for high).

From this multi-modal state, the agent can chose the following actions: sending an order to the order book $a_{0}^{T}$ (equal to 0 for shorting, 1 for holding, 2 for longing), and at what price above or below the agent’s own price estimate $a_{1}^{T}$ (equal to 0 for indifference to lose on transaction, 1 for neutral, 2 for willingness to gain on transaction) via the transaction gesture *g*^*i*^ scaled with the market spread *W*^*j*^(*t*).

Considering the present stock price *P*^*j*^(*t*), the algorithm $T^{i}$ then computes the cashflow difference between the agent’s portfolio net asset value, and its present value if the former actions taken *τ*^*i*^ time steps ago had not been taken: $Q_{\text{OB}}^{i,j}\left(t-\tau^{i}\right)\left[P^{j}\left(t\right)-P_{\text{OB}}^{i,j}\left(t-\tau^{i}\right)\right]$. Here $Q_{\text{OB}}^{i,j}\left(t-\tau^{i}\right)$ and $P_{\text{OB}}^{i,j}\left(t-\tau^{i}\right)$ are respectively the quantity and transaction price of stock *j* that was cleared by the order book process at time *t* − *τ*^*i*^ for agent *i* and its transaction partner. Notice these may not be those actually sent by agent *i* at that time, because the quantity of stocks to long or short may not have been entirely cleared at this time (recall the agents send limit orders only), and because the transaction price is set by the order book at mid-price with the transaction partner’s order price.

Same as for the algorithm $F^{i}$, these values are then recorded at each time step in a time series that is continually sorted in ascending order and truncated to keep a size corresponding to agent memory interval *h*^*i*^. The associated percentile corresponding to this value at time step *t* sets a discrete value of returns $r^{T}$ in the set {4, 2, 1, −1, −2, −4} if it respectively belongs to the intervals [0%, 5%(, [5%, 25%(, [25%, 50%(, [50%, 75%(, [75%, 95%(, [95%, 100%].

The rewards of these performed actions are defined via the difference in cashflow at time *t* between the profit or loss consequent to the agent’s past action at time *t* − *τ*^*i*^, and the one had this action not been taken. Again, these feed a direct policy update with new action probabilities for the agent in such a state.

Our MAS model make a number of simplification over several aspects of real financial markets. First it assumes homogeneity among agents in terms of their learning algorithms and market impact, which might not accurately represent the diverse investor profiles in actual markets. Secodn, this study’s focus on learning rate, herding, and noise trading at the agent level does not encompass other potentially influential factors such as regulatory changes, macroeconomic indicators, or global events, which play a significant role in real-world financial markets. In the section 8.2.5 of the Supplementary Material, we give more details on the limitations of our model.

## 4 Agent learning rate increase impacts on market crashes

**Fig 2:**
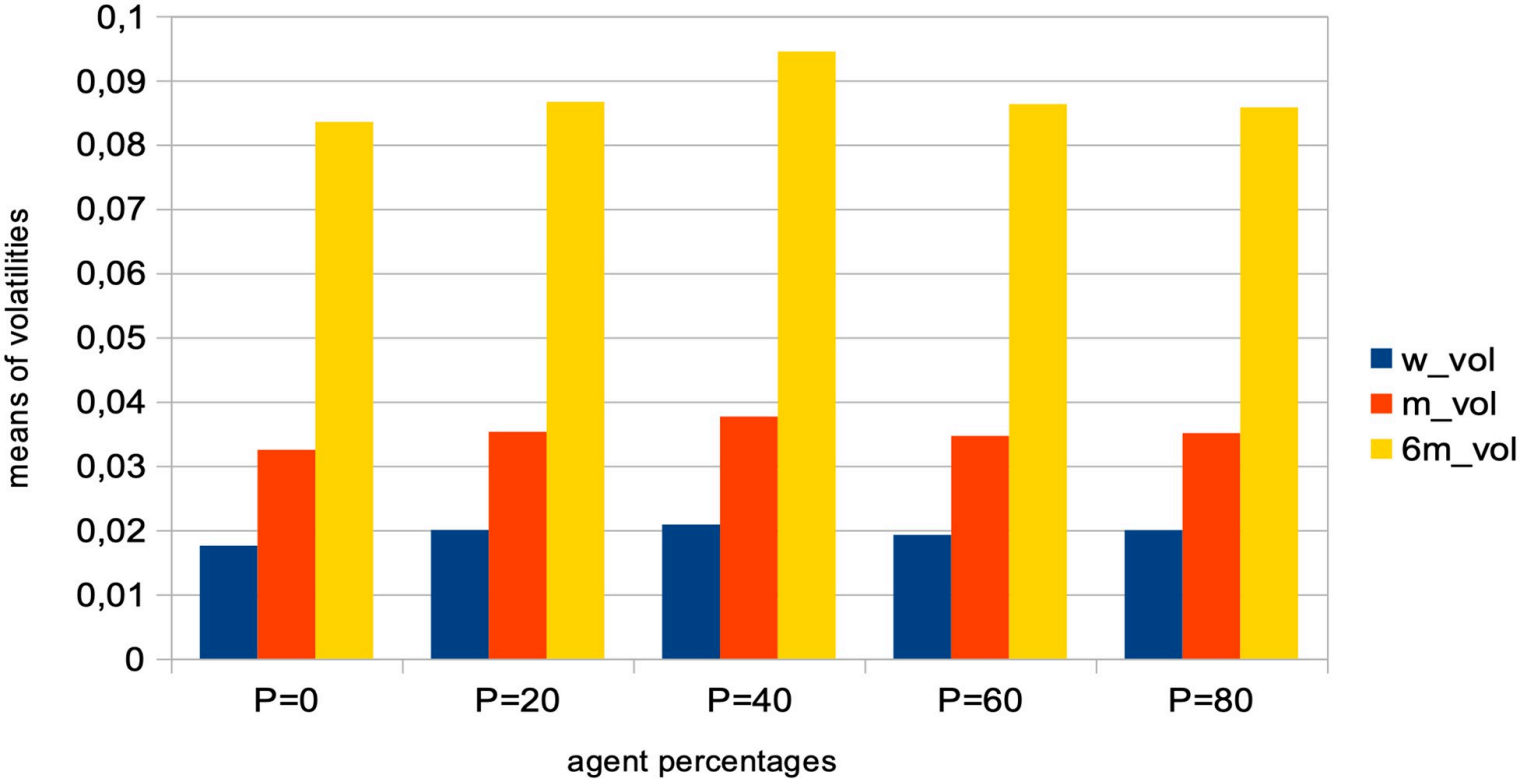
Means of various price volatilities for percentages of agents with a doubled learning rate. Means of volatilities, defined as the standard deviations of price normalized to the price itself $\frac{\sigma }{P(t)}$, computed over different time lags: one week (blue), one month (red), and six months (yellow) intervals. These are calculated for varying percentages *p* of agents, corresponding to *p* = 0%, 20%, 40%, 60%, 80% of the total agent population. Agents in these percentages have their learning rate doubled, while the remaining 100 − *p*% of agents maintain the usual learning rate, with β∼U(0.05,0.20). Results are derived from *S* = 20 simulation runs, involving *I* = 500 agents, *J* = 1 stock, over a period of *T* = 2875 time steps.

**Fig 3:**
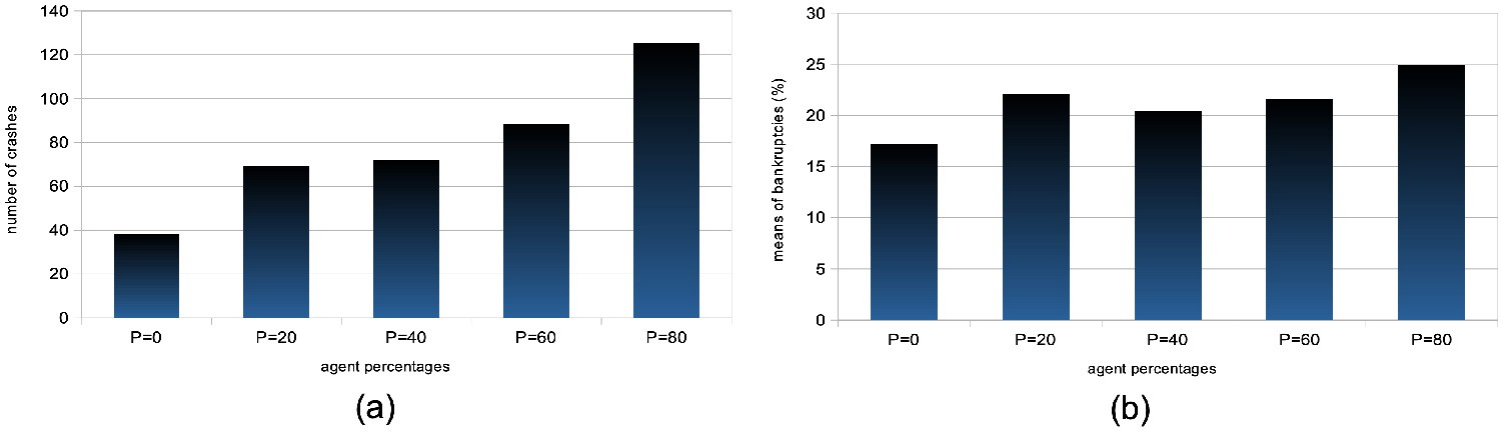
Means of crashes and bankruptcy rates for percentages of agents with a doubled learning rate. For both plots (a) and (b), varying percentages *p* = 0%, 20%, 40%, 60%, 80% of the total agent population were analyzed, where agents had their learning rate scaled by a factor of 2 and the remaining 100 − *p*% had a learning rate *β* drawn from U(0.05,0.20). These analyses come from *S* = 20 simulation runs with *I* = 500 agents, *J* = 1 stock, over *T* = 2875 time steps. (a) Examines the mean number of market crashes, defined as a 20% decrease in asset price at time *t* + 1, across different *p* values. (b) Calculates the mean percentages of bankrupt agents, with bankruptcy defined as an agent’s drawdown reaching its limit *l*^*i*^, for varying *p* values.

We first focused on assessing how the reinforcement learning rate influences agent performance and the broader market dynamics. This was in large part motivated by observations in behavioral neuroscience that identified learning rate differences as a key factor in the heterogeneous performance on value-driven decision tasks in humans. We thus gauged how inserting into the simulated agent population a progressively larger proportions of agents with a statistically larger learning rate would change the overall market quality. Changing the agents’ learning rate amounts to varying their sensitivity to their most recent observed outcomes. Here *most recent* reflects a time range between one week up to six months worth of trading, given by the *τ* range. We thus statistically doubled the learning rate *β* in a increasing fraction *p* of the total agents (i.e. *p* = 0%, 20%, 40%, 60%, 80%). Recall each agent is initialised with a learning rate modelled by the parameter β∼U(0.05,0.20) for both reinforcement algorithms $F^{i}$ and $T^{i}$. Simulations of trading in our ABM under such variations in quantity *p* of the proportions of agents with doubled learning rates, lead us to observe the following impacts at the market level:

- Price volatility: We see that price volatilities remain stable at different time-scales, for variations in quantity *p* (see Fig 2). A structural explanation for this is that agents with larger learning rates send transaction orders with prices that more closely reflect recent microstructure variations, hence providing more liquidity to the market.
- Market crashes: We see that the number of market crashes increases strongly (see Fig 3a). This is an expected result, as larger learning rates imply agents that will amplify the latest market tendencies reflected in the microstructure. Notice the standard definition of a crash is here applied, namely a decrease in 20% of the asset price at time *t* + 1.
- Agent bankruptcy: We find that percentages of agent bankruptcies barely increase (see Fig 3b). This should be here understood in the light of the two previous conflicting factors wrt. market stability, namely that the price volatilities remain stable for such agents, and at the same time that the market experiences a larger amount of crashes due to amplified market tendencies, and we posit the latter to cause the slight increase on the plot of Fig 3b.

It is thus interesting to note that such variations in agent proportions with doubled learning rates in our model do not affect general market volatility, except in tail events with statistically much greater numbers of market crashes,. We also note that that the mean numbers of agent bankruptcies are mildly affected. It is interesting to put these results in the context of the hypothesis on the adaptability of marketsAccording to the Adaptive Market Hypothesis [75] (AMH), stock markets can exhibit both efficiencies and inefficiencies concurrently because market participants do not operate solely on irrational or rational behavior, but will potentially adapt and make decisions informed by their past experiences. The AMH hence suggests that market participants adapt their strategies based on changing market conditions. Rapid learners (i.e. agents with larger learning rates) might adapt quickly to new trends, while slow learners may stick to traditional strategies. We posit that exploring further the interplay between our results and the proposed factors in the context of the AMH in traditional economics will be a fruitful direction to pursue in the future.

## 5 Impact of agent herding

We next considered the impact of agent herding on the market dynamic. Notably, we hypothesized that agent herding may have a crucial impact on market stability as it is related to market reflexivity [57] (i.e. market endogeneity). As we pointed out in Section 1, an intuitive way to model agent herding is to set populations of agents that follow the trading or investment advice of another “modal” agent, based on investment information (recommendations or reviews) broadcast to the agent population.

In our framework, we model the impact of such agent herding on the market as a whole by introducing increasing percentages *p* of agents sending (when possible) the same transaction order to the order book at time *t* + Δ (for $\Delta \in N^{+}$) that was sent by the agent with best trading performance or track record at time *t*. For the sake of simplicity, we here consider this “best” agent as the one with the largest net asset value at time *t*. Hence, the rest of the herding agents may follow and emulate different agents over time, just as in real markets. Also, because of the different trading horizons $\tau^{i}∼U\left\{T_{w},6T_{m}\right\}$ of the agents, we narrow our study here below to the simplest case and set Δ = 1.

**Fig 4:**
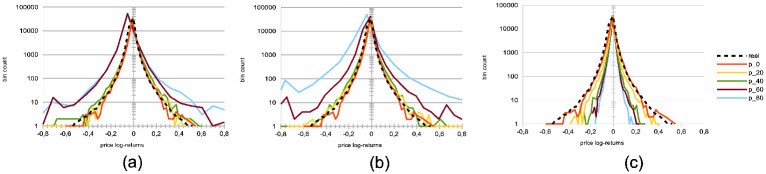
Distributions of price log-returns for different percentages of agents imitating the best agent, worst agent, and engaging in random trading. We analyze the distribution of logarithmic returns of real and simulated prices, denoted as log[*P*(*t*)/*P*(*t* − 1)]. The real price data is represented by a dashed black curve, while the simulated data is depicted by continuous curves. The simulations correspond to different percentages *p* of agents, where *p* = 0%, 20%, 40%, 60%, and 80% of the total agent population at time *t*. These percentages are represented by different colors: red (*p* = 0%), yellow (*p* = 20%), green (*p* = 40%), brown (*p* = 60%), and light blue (*p* = 80%). The agent behaviors are categorized into three types: (a) following the best agent, (b) following the worst agent, (c) trading randomly, while the remaining 100 − *p*% of agents engage in proprietary trading strategies. These results are derived from *S* = 20 simulation runs, involving *I* = 500 agents, *J* = 1 stock, and spanning *T* = 2875 time steps.

**Fig 5:**
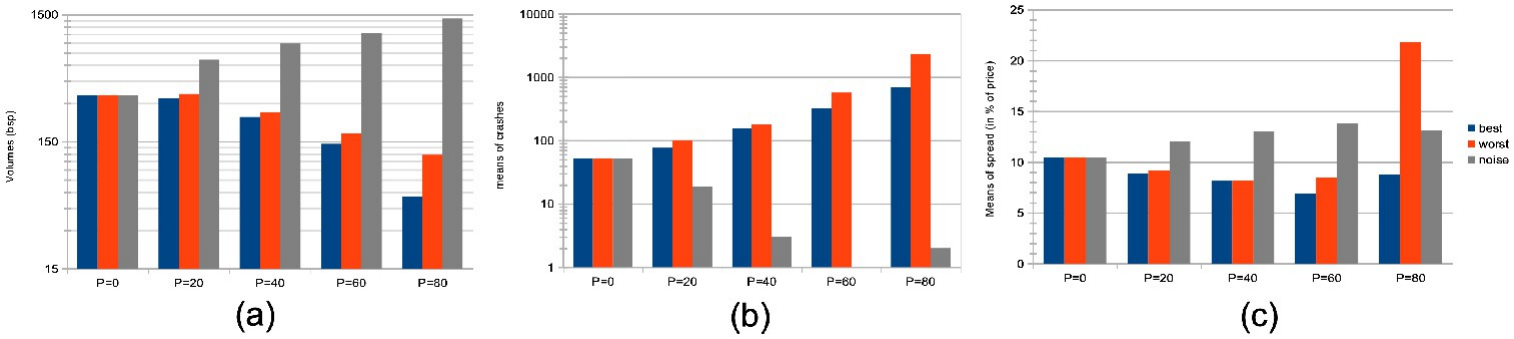
Means of volumes, numbers of crashes, and spreads for different percentages of agents imitating the best agent, worst agent, and engaging in random trading. Figures (a), (b), and (c) present aggregated results from *S* = 20 simulations with *I* = 500 agents and *J* = 1 stock over *T* = 2875 steps. In (a), we calculate the mean trading volumes for agents partitioned into percentages *p* = {0%, 20%, 40%, 60%, 80%} of the total, divided by strategy: best-following (blue), worst-following (red), random (grey), and proprietary strategies for the remaining 100 − *p*%. In (b), we depict the mean number of market crashes—defined as price drops greater than 20% at *t* + 1—for the same distributions of *p*, categorized by the same strategies. (c) showcases the mean bid-ask spreads, as a percentage of price, for varying *p*, with agent behaviors similarly categorized.

In order to gauge the results of our study, we compare the resulting market dynamics with simulations where increasing percentages of the agents follow the agent with the *worst* trading performance at time *t*. Again, we consider this “worst” agent as the one with the lowest, non-bankrupt, net asset value at time *t*. These two approaches (that we will call “best agent herding” and “worst agent herding” respectively) taken together allow us to qualitatively gauge the market impact from the agents herding a best agent in a statistically robust manner. Results are shown on Figs 4 to 5c, and allow us to make the following conclusions:

- Both herding scenarios increase the market price volatility. The “best agent herding” leads to a strong increase in price volatilities. This effect especially clear for larger herding percentages (see Fig 4a). At the same time, a market with “worst agent herding” displays an increase in price volatilities that is even stronger than the previous case, especially for larger percentages of herding agents (see Fig 4b). This market also shows a significant skewness of the return distribution in favour of larger negative returns. We note this volatility increase for both distributions is almost imperceptible for cases where the percentage of herding agents stays below half of the total agent population (*p* < 50), and also that the average returns are negative for *p* > 50. One could understand this result by the fact that in both scenarios for *p* > 50, more agents will send orders to the order book that will rarely find a matching order for a transaction to be validated. Together with the results shown in the plots of Fig 5a–5c below, we posit this can produce a number of transaction fails and illiquidity issues in the agents’ portfolio that yield negative market returns at the mesoscale.
- Trading volumes decrease drastically in both herding scenarios, albeit much more so for the “best agent herding” case (see Fig 5a, blue curves). As said previously, the a straightforward explanation for this loss of market liquidity with larger populations of both “best” and “worst” herding agents is that increasingly more agents send transaction orders finding no match within the order book. This order book hence becomes filled with one-sided short orders mostly, or long orders mostly, depending on the “best” or “worst” agent’s own trading order at time *t* − 1, with little to no orders matching them for actual transactions, thus reducing the whole market trading volumes.
- Market crashes increase exponentially in both scenarios, but even more so for the “worst agent herding” case (see Fig 5b, red curves). This can be explained from the decrease in trading volumes for larger values of *p* in both scenarios, since stock illiquidity prevents agents to obtain transactions matching their orders, and hence manage their portfolio accordingly.
- Market bid-ask spreads decrease steadily, until *p* > 60%, after which they slightly increase again for the “best agent herding” scenario, and nearly double for the “worst agent herding” scenario (see Fig 5c). This may seem contradictory with the previous results about illiquidity and exploding numbers of crashes. Indeed, it is known that the more disagreement on stock pricing between agents, the more trading activity and volumes, as well as price volatility [76]. Apart from the “worst agent herding” scenario with *p* = 80%, one should notice this steady decrease in spreads is quite mild, and hence cannot account by itself for the aforementioned effects on the market.
- Remarkably, the rates of agent bankruptcy remain stable regardless of these varying percentages for the “best agent herding” scenario, with average means of 22.76 ± 3.25% for all values of *p*. As for the “worst agent herding” scenario, the rates of agent bankruptcy greatly increase with these varying percentages, staying above 70% of agent bankruptcy for *p* > 20%, as one could expect.

These results may be counter-intuitive at first, however, we see that passively following a renowed investor (defined as the agent with the largest net asset value at time *t*) according to this “best agent herding” scenario (defined by *p* agents emulating such an investor transaction order at time *t* + 1) is extremely averse to market stability, notably in terms of negative price returns, increased illiquidity and numbers of crashes.

We would like to note that from the traditional economics point-of-view, herd behavior may not be necessarily irrational, and may actually be compatible with optimizing behavior [77]. A greater trust in collective, rather private, information may be a rational choice in case of information asymmetries or imperfect information, where the trader leverages collective information processing. While this approach may be seen as rational from the individual point of view, it may be detrimental at market level (on the lines of the tragedy of the commons), breaking the resilience of this strategy exploiting collective intelligence. Herding can otherwise be seen as group pressure [78, 79], social conformity or a heuristic based on self-amplifying noisy information. In the past, herding behaviour has been studied as “information cascades” [78], though in these models individual decisions occurred sequentially. Subsequent models dropped this unrealistic assumption, while focusing on the different sources of information to consider. In terms of heterogeneity of imitation, our study stands between [80], where all agents imitate each other at the same extent while mixing private information, and [81], where imitation occurs in random independent groups, from a random communication structure between agents. In line with the latter study, where imitation gives rise to heavy tails, in our model, extreme events (market crashes) increase as herding behaviour increases.

## 6 Impact of noise traders

**Fig 6:**
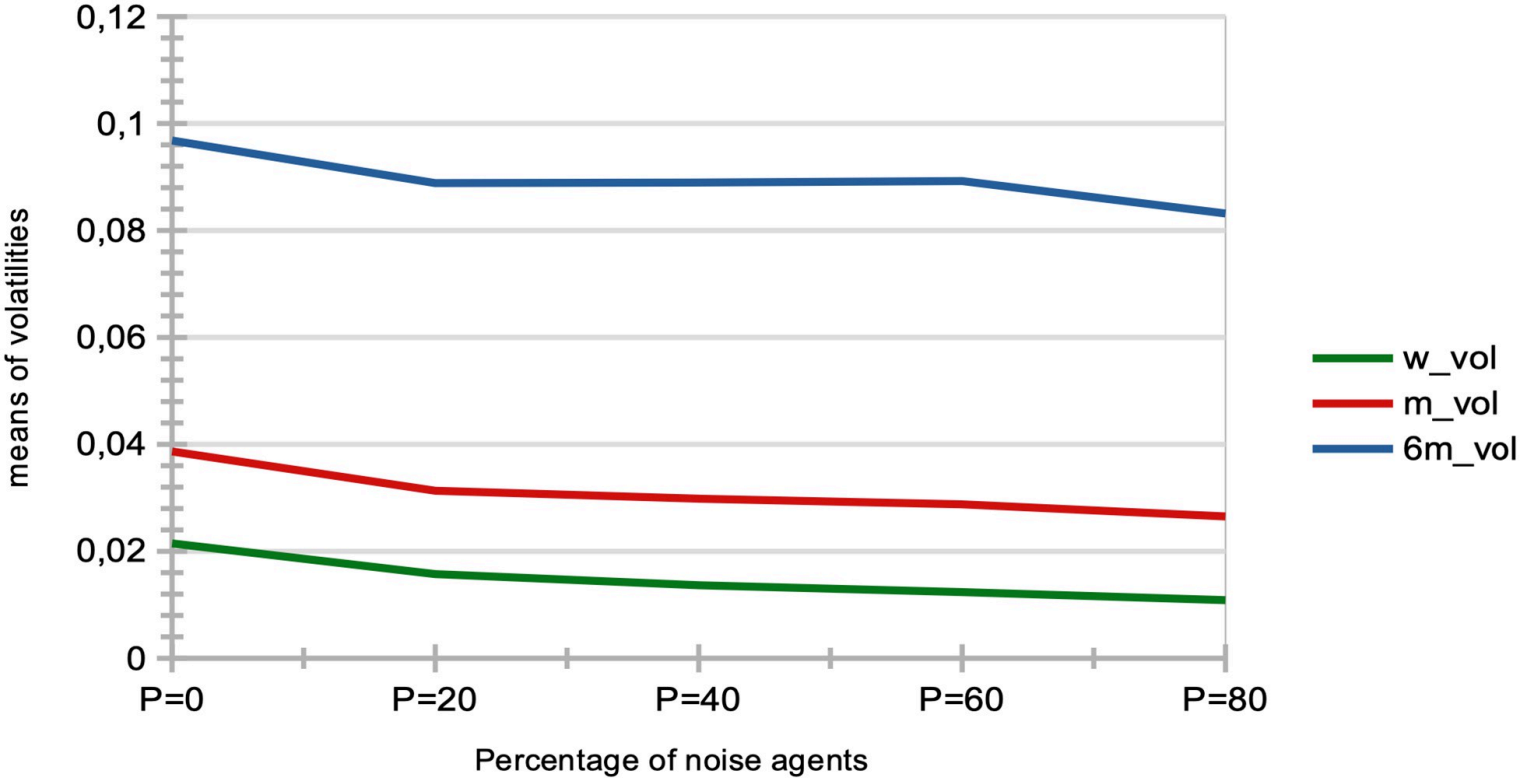
Means of various volatilities for different percentages of agents engaging in random trading. Means of volatilities, defined as the standard deviations of price normalized to the price itself $\frac{\sigma }{P(t)}$, were computed over various lags: one week (green), one month (red), and six months (blue). These computations were carried out for different percentages of agents, *p*, corresponding to *p* = 0%, 20%, 40%, 60%, 80% of the total agent population engaged in random trading. The remainder of the population, 100 − *p*, participated in proprietary trading strategies. These results were derived from *S* = 20 simulation runs, generated with *I* = 500 agents, *J* = 1 stock, and conducted over *T* = 2875 time steps.

**Fig 7:**
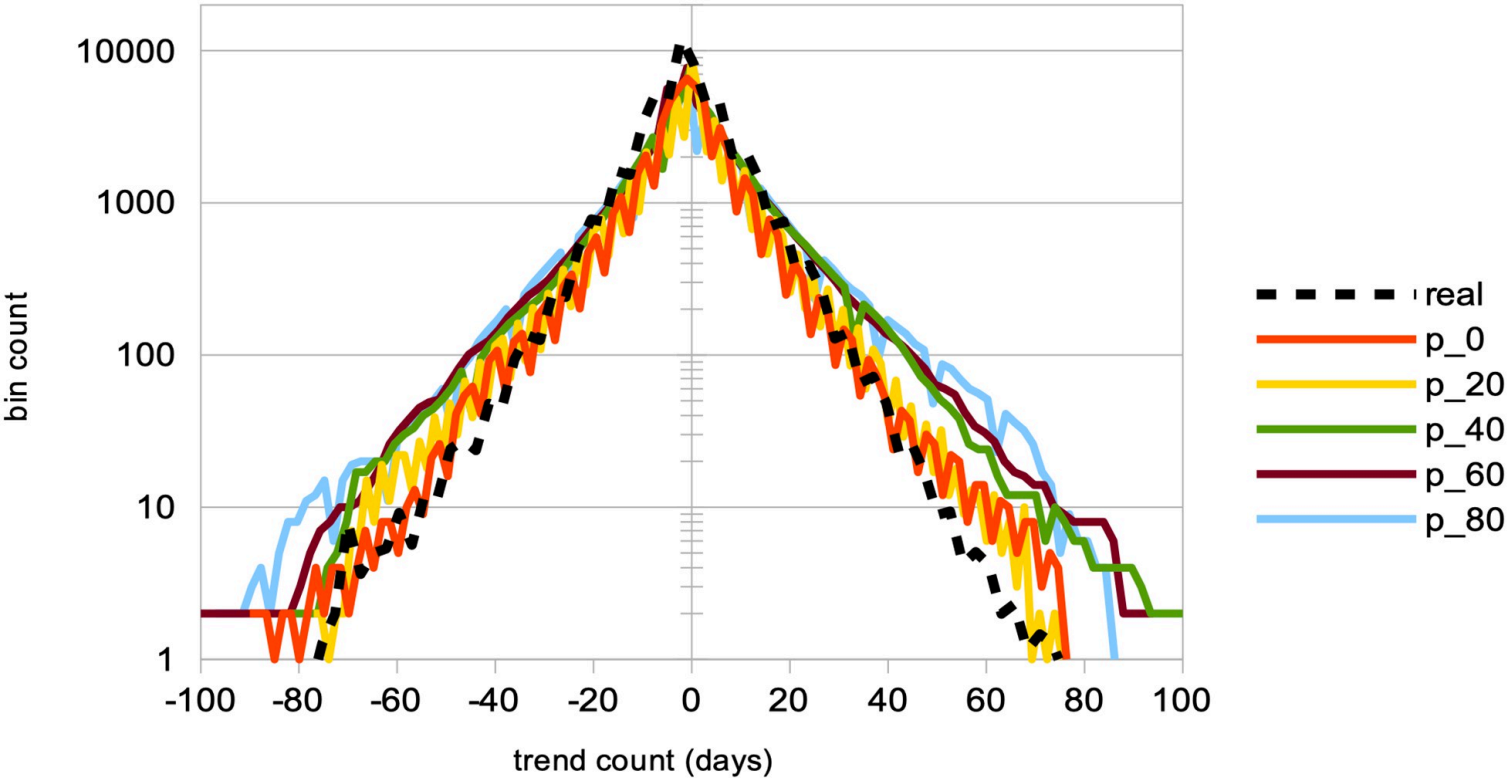
Distributions of the consecutive days of bull and bear market regimes, for different percentages of agents engaging in random trading. The distribution of the number of consecutive days of rising prices (positive values) and dropping prices (negative values) is analyzed. This analysis includes both real data (represented by a dashed black curve) and simulated data (represented by continuous curves). The simulated data corresponds to varying percentages of agents, *p*, engaging in random trading, while the remainder (100 − *p*%) employ proprietary trading strategies. Specifically, the distributions for *p* = 0% (red curve), *p* = 20% (yellow curve), *p* = 40% (green curve), *p* = 60% (brown curve), and *p* = 80% (light blue curve) are shown. These results are derived from *S* = 20 simulation runs, involving *I* = 500 agents and *J* = 1 stock, conducted over *T* = 2875 time steps.

Our next objective was to explore the mesoscale effects of a rising proportion of ‘noise traders,’ here defined as agents engaging in random trading [59], so as to check on the role played by agent information on market price formation [58]. Following the efficient-market hypothesis [65, 66]), we hypothesised that an increasing number of noise agents should bring a certain financial stability to the whole market, by providing more liquidity and higher trading volumes in both bid and offer. This is predicted since an efficient market of sufficient size would ensure market prices at their fair value, and no profit consistently earned over time by investors buying undervalued stocks and selling overvalued ones, or exploiting historical data patterns so as to forecast future data. With increasing percentages of “noise agents” *p*, we observe the following results:

- A strong decrease in the absolute value of logarithmic price returns (see Fig 4c). This is congruent with what we see also on Fig 6, which displays a general decrease in means of price volatilities computed over several time scales (namely, lags of one week, one month, and six months), for a percentage *p* of agents corresponding to *p* = 0%, 20%, 40%, 60%, 80% of the total agent population trading randomly (the remainder 100 − *p* engaging in proprietary trading strategies). This decrease in price returns and volatilities with larger values of *p* can be explained by the larger diversity of orders sent by the agents to the order book, and hence the greater opportunity for these orders to find matching orders. This is confirmed by the following result about trading volumes.
- A very strong increase in trading volumes for increasing proportions of noise agents (see Fig 5a). One could explain this strong decrease by the same reasons as previously.
- A sharp decrease in market crashes, which virtually almost vanish for *p* > 50% (see Fig 5b). As previously, this can be explained by the greater number of both short and long orders due to the random trading of the agents: sharp increase or decrease in prices (i.e. crashes) are hence less likely for larger values of *p*.
- A steady increase in bid-ask spreads (see Fig 5c). A reason for this would again be that more diverse orders are sent to the order book with larger proportions of noise traders, and hence that the spread less likely shrinks because of a lack of matching orders, as both sides (i.e. short and sell order types) of the order book are more likely populated with increasing values of *p*.
- A steady increase in length of both bull and bear market regimes (see Fig 7), resp. defined as the number of consecutive days of rising prices (positive values) and dropping prices (negative values). We see especially strong effects on the bull market regime lengths. This is an interesting result, as one could have expected the larger amount of liquidity provided on both sides of the order book for increasing values of *p* would imply a more versatile market microstructure, despite the associated decreasing volatility.
- Finally, agent bankruptcy rates steadily decrease with higher proportion of noise traders, from means of 23.22% for *p* = 0%, to 18.84% for *p* = 80%. This is remarkable, as one could have posited that agent survival rates would decrease because of such random trading. One should link this result to other related studies probing the performance of random asset management [82].

In line with other recent studies [83], we hence conclude that counter-intuitively, larger numbers of agents trading randomly is beneficial to market stability and even to agent performance. Under larger proportions of such noise trading agents, we see that market price volatilities decrease, trading volumes strongly increase, market crashes virtually vanish, and agents bankruptcy slowly decrease. While random trading would be the only meaningful trading in a fully efficient market, the emergence of “inefficiencies” in a market of traders employing well-informed trading strategies is in line with the reasoning that noise traders are the ones providing other traders the profit opportunities necessary to have a market [75]. Quoting Black [84]: “*Noise makes financial markets possible, but also makes them imperfect*.” Our results therefore contrast with others of classical economics such as [85], who instead argue that the market structure is sufficient to establish full efficiency even when traders behave randomly, leading the authors to conclude that “*imposing market discipline on random, unintelligent behavior is sufficient to raise the efficiency from the baseline level to almost hundred percent in a double auction. The effect of human motivations and cognitive abilities has a second-order magnitude at best*”.

## 7 Discussion

In this work we presented a bottom up exploration of the mesoscale impacts of trader learning behaviors in financial markets by using a multi-agent reinforcement learning model. This approach provides for a better understanding of how individual trader characteristics, such as learning rate amplification, herding behavior, and noise trading, collectively influence market dynamics. This also allowed us to explore how individual trader decisions interplay with broader market phenomena, with a different angle than other classical top-down economic models. By bridging the gap between micro-level trader behavior and macro-level market outcomes, both regulatory bodies and financial practitioners can leverage these insights for more robust market predictions and strategies in an increasingly complex financial landscape.

The “SYMBA” MAS stock market simulator we used for this work [53, 54], was calibrated to the London Stock Exchange data between the years 2007 and 2018. In this model, the agents autonomously manage their portfolio via a long-only strategy based on two reinforcement learning algorithms: one performing price forecasting and another one performing stock trading. In SYMBA, each agent is also endowed with specific and relevant reinforcement learning parameters that allow us to quantitatively study the impact of agent learning on financial stock markets at the mesoscale.

Table 1 gives an overview and general sum up of the results of our experiments. We first studied the impact on the market of increasing proportions of agents with doubled learning rates. We found that market price volatilities at all time-scales did not vary much. A structural explanation for this is that agents with larger learning rates send transaction orders with prices more closely reflecting recent microstructure variations, hence providing more liquidity to the market. We further found average numbers of crashes to greatly increase. This is an expected result, since larger learning rates imply agents that will amplify the latest market tendencies reflected in the microstructure. We also found that agent bankruptcy rates were not much impacted by variations in agent populations with doubled learning rates. This underscores the influence of learning rates on market stability, and highlights the balance between rapid adaptation and potential market volatility.

**Table 1:** General sum up of the key results of the study.

Cognitive Trait	SYMBA Implementation	Market Impact
**Larger agent learning rate**	Larger percentages *p* of agents with a doubled agent learning rate	Stable price volatilities (Fig 2); Increase of market crashes (Fig 3a); Stable rates of agent bankruptcies (Fig 3b).
**Impact of agent herding**	Larger percentages *p* of agents imitating the best performing agents at time *t*	Increased price volatilities & larger negative returns (Fig 4a), esp. for *p* > 50%; Much larger trading volumes (Fig 5a); Drastic increase in market crashes (Fig 5b); Bid-ask spreads decrease for *p* < 60% and increase for *p* ≥ 60% (Fig 5c); Stable rates of agent bankruptcies.
**Impact of agent herding**	Larger percentages *p* of agents imitating the worst performing agents at time *t*	Greatly increased price volatilities & larger negative returns (Fig 4b), especially for *p* > 50%; Much smaller trading volumes (Fig 5a); Exponential increase in market crashes (Fig 5b); Bid-ask spreads decrease for *p* < 60% and strongly increase for *p* ≥ 60% (Fig 5c); Strong increase in agent bankruptcy rates.
**Impact of noise traders**	Larger percentages *p* of agents trading randomly	Strong decrease in absolute log-price returns & steady decrease in price volatilities (Figs 4c and 6); Explosion of trading volumes (Fig 5a); Sharp decrease in market crashes (Fig 5b) which almost vanish for *p* > 50%; Bid-ask spreads steadily increase (Fig 5c); Longer bear and esp. bull market regimes (Fig 7); Steady decrease in agent bankruptcy rates.

We then studied the effect of agent herding, when increasing percentages of agents followed and emulated at time *t* + 1 the investments of the best performing agent at time *t*, and found that, as expected, such a herding behaviour greatly increases market instability, with an increase in negative market price returns, illiquidity and numbers of crashes. Yet, remarkably, bankruptcy rates of simulations with greater amounts of agents imitating a top-performer remain quite stable, regardless of the percentages of such agents, and regardless of these increasing market volatilities and numbers of crashes. The study of herding behavior thus revealed its profound impact on market stability. Markets where a significant percentage of traders emulate the investment choices of top-performing agents exhibit increased negative price returns, illiquidity, and frequency of crashes.

Finally, keeping in mind the ever-increasing amount of information and big data available to practitioners, and its role in price formation, we sought to explore the impact of agent information and rationalityon the price formation process and so-called market memory, with larger proportions of “noise traders” (i. e. agents trading randomly). We found a much greater market stability with increasing percentages of such agents, with strongly decreasing price volatilities at all time scales, and a number of crashes virtually vanishing. Economically, this can be explained by the larger diversity of orders sent by the agents to the order book, and hence the greater opportunity for these orders to find matching orders, thereby avoiding illiquidity issues. We also found such markets to be more prone to display bull regimes, and that agents bankruptcy rates slightly diminished. The result that market stability is enhanced by random trading and agent irrationality may be a challenge to traditional views on market dynamics and rational trading behavior such as the Efficient Market Hypothesis.

The implications of these findings may be significant for both regulatory policies and investment strategies. By understanding the nuanced effects of individual trader behaviors and collective dynamics, regulatory bodies can develop more effective oversight mechanisms, and financial practitioners can refine their market strategies to better adapt to an increasingly complex financial landscape. The mesoscale perspective adopted in this study also bridges the gap between micro-level behaviors and macro-level outcomes, providing a comprehensive view of market dynamics. Future research could extend these findings by exploring the influence of other trader characteristics and market conditions, in order to enrich our understanding of financial markets in the modern economy. Another natural extension of this research would be to study the mesoscale impact of the order book parameters, and see how this would change the policies learned by the agents.

With a broader view, the relevance of the MAS approach is also reinforced by the fact that financial markets today are a rapidly evolving technological landscape. By studying human traits of behavior in a quantitative way, the MAS approach can help better understand the potential impacts of automated trading systems wrt. risk management and systemic risk, and how these systems behave under different market conditions influenced by rapid technological advancements.

## 8 Supplementary material

### 8.1 Primer on reinforcement learning

*Overview*: We here briefly review the basics of reinforcement learning theory that pertain to this study. Together with supervised and unsupervised learning, reinforcement learning has been termed one of the three paradigm shifts of machine learning [68], and is today at the forefront of almost all breakthroughs in machine learning research. Like many other machine learning methods, reinforcement learning has its roots in behavioural psychology and decision theory [69]. In reinforcement learning, we consider an agent in a given *state* of its environment that must learn the best way to consistently receive a preset *reward* from its environment through a selection of its possible *actions*. The whole reinforcement learning problem and solution is thus how the agent selects these actions in a dynamic environment so as to maximise this reward. In the beginning of the task, the agent is completely agnostic as to which actions are best to use: it will have to learn this on its own.

**Fig 8:**
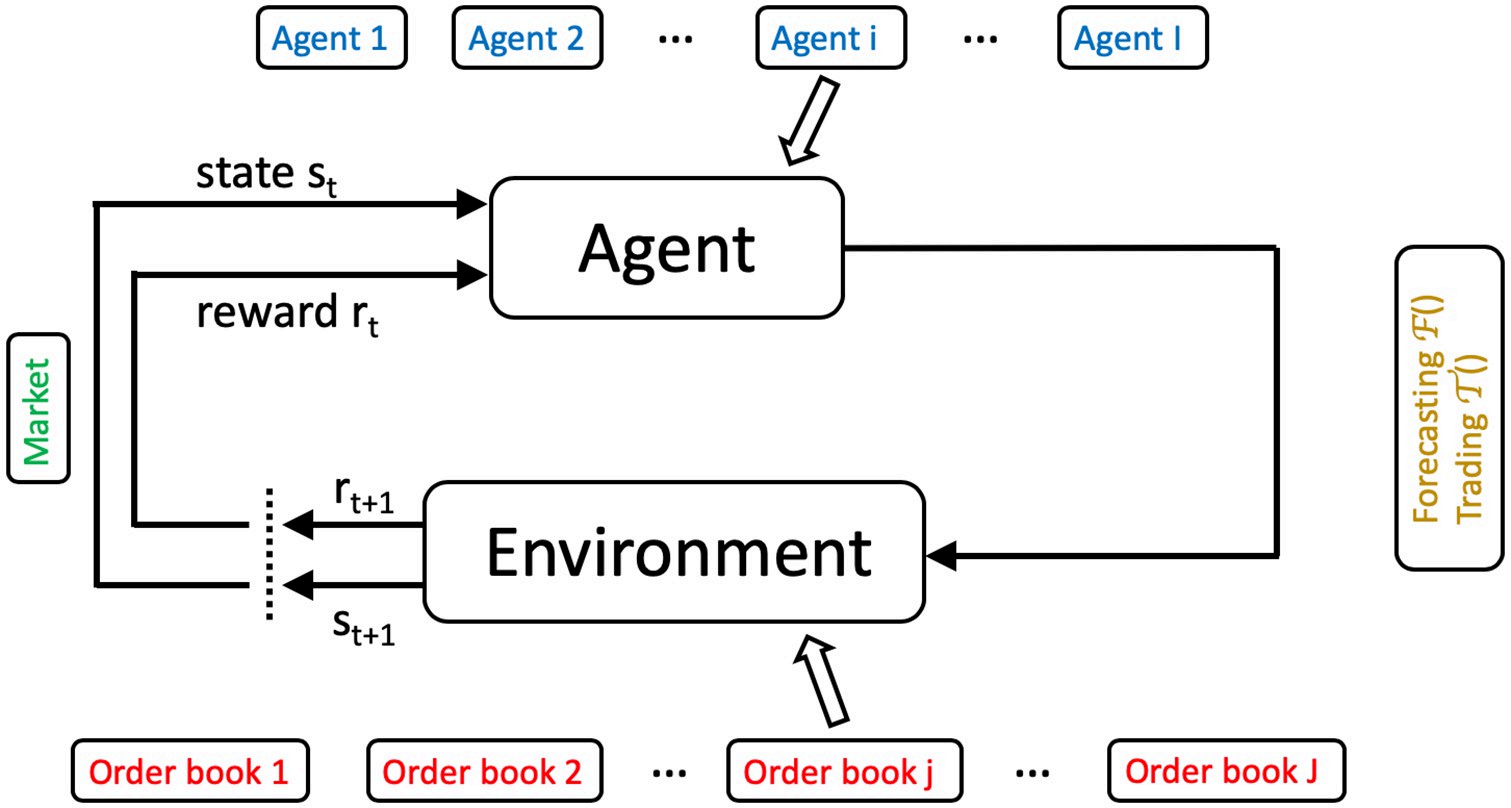
Schematic of the reinforcement learning procedure. Classical algorithmic procedure of a reinforcement learning agent at time step *t* in the context of SYMBA described below. In a given state *s*_*t*_ of its environment (i.e. the market), a given agent *i* selects one of its actions *a*_*t*_ (from its forecasting or trading algorithm) with respect to the market order book of a given stock stock *j*, thus yielding an associated given reward *r*_*t*+1_ and new state of the environment *s*_*t*+1_.

*Parameters*: The reinforcement learning problem is thus defined with three main parameters: the states of the environment s∈S, the agents actions a∈A, and the agent reward r∈R. The basic iteration procedure of reinforcement learning is shown on Fig 8. The states and actions can be defined as more or less complex concepts, and notice the rewards can be positive or negative. The goal of reinforcement learning for the agent is to find its policy *π*(*s*, *a*) = *Pr*(*a*|*s*), which is the set of probabilities associated to each of the agent’s action selection within each possible state of its environment, so as to maximise its rewards. In order to do this, three major types of reinforcement learning algorithms are used: i- model-based methods rely on the agent estimating two functions called the transition probability $P_{ss^{\prime }}^{a}=Pr\left\{s_{t+1}=s^{\prime }|s_{t}=s,a_{t}=a\right\}$ and the expected value $R_{ss^{\prime }}^{a}=E\left[r_{t+1}|s_{t}=s,a_{t}=a,s_{t+1}=s^{\prime }\right]$, where 0 < *γ* < 1 is a discount parameter related to the concept of delayed reward, and out of these derive the so-called *state-value function*: $V\left(s\right)=E\left[\sum_{k=0}^{\infty }\gamma^{k}r_{t+k+1}|s_{t}=s\right]$. ii- Model-free methods rely more simply on the estimation of the so-called *action-value function* $Q\left(s,a\right)=E\left[\sum_{k=0}^{\infty }\gamma^{k}r_{t+k+1}|s_{t}=s,a_{t}=a\right]$. These functions *V*(*s*) and *Q*(*s*, *a*) in model-based and model-free methods thus allow the agent to update its policy *π*(*s*, *a*), which in turn shall be used at the next time step of the task to select a relevant action *a*, and iteratively proceed in a same manner so as to hopefully converge to an optimal policy denoted π*(*s*, *a*). iii- Policy-based methods directly update the policy *π*(*s*, *a*) = *Pr*(*a*|*s*) according to the returns received from the environment, following the agent action selection. This update is performed according to Eqs 1 and 2.

*Features*: Three major features appear here, and are at the centre of most if not all reinforcement learning research: i- a *curse of dimensionality* arises from the number of state-action pairs, since if these are two numerous, the problem of convergence to a policy may be intractable. ii- A *temporal credit assignment* is another issue pertaining to how rewards are practically defined for the task at hand, and how the temporal discounting of these rewards is set. iii- An *exploration vs. exploitation* dilemma is another important feature of reinforcement learning, which pertains to whether it is profitable for the agent to exploit the rewards linked to a good policy it found in its environment, or whether it is better to continue exploring and (perhaps) attain to a better policy and hence rewards.

### 8.2 Model architecture

We here outline the structure of our stock market Multi-Agent System (MAS) simulator and the design principles behind its autonomous agents, in more details.

#### 8.2.1 Architecture overview

**Fig 9:**
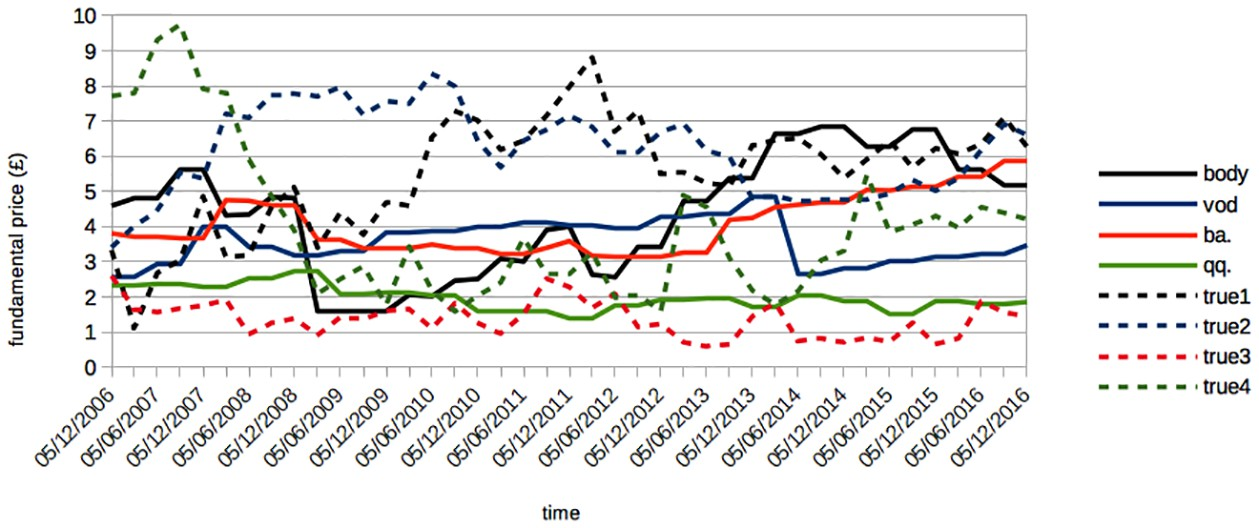
Enterprise values per number of stocks outstanding vs. their associated prices for several LSE stocks. Illustration of key metrics for select entities on the London Stock Exchange (abbreviated as follows: Bodycote plc—“body”, Vodafone Group plc—“vod”, Boeing co.—“ba.”, QinetiQ Group plc—“qq”) from the period 2006 to 2016. This figure shows the enterprise value per outstanding share (depicted as solid lines) and *J* = 4 non-scaled temporal series $T^{j}\left(t\right)$ (shown as dotted lines) produced by our simulation at the initial time point *t* = 0.

**Fig 10:**
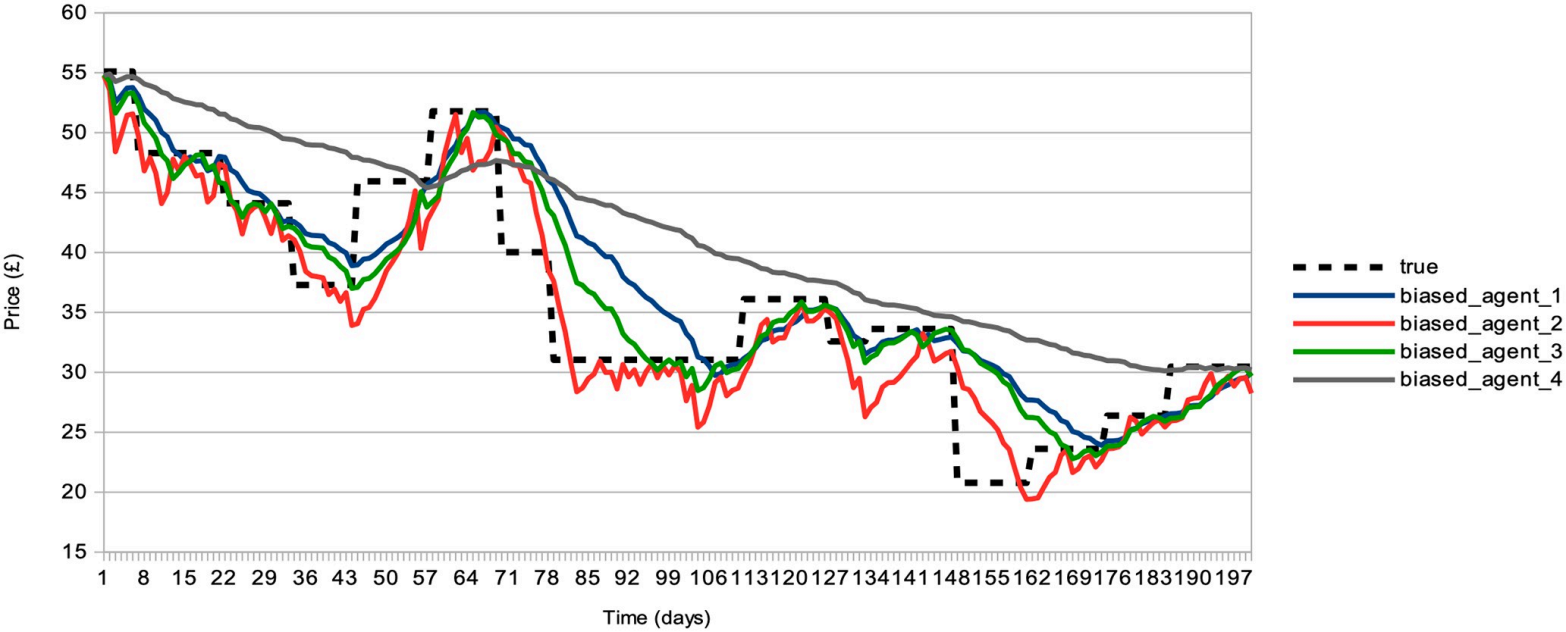
Fundamental values vs. agents’ biased values. Depiction of fundamental value trajectories modelled by $T^{j}\left(t\right)$ (represented as a dashed black line) and their perceived estimates $B^{i,j}\left(t\right)$ by four different agents (shown as solid lines in blue, red, green, and gray), across a simulated duration of 200 time steps.

As already said in Section 3, the key parameters defining our simulation include: the total number of agents, denoted as *I*; the quantity of stocks traded, indicated as *J*; and the duration of the simulation in time steps, represented as *T*. Here, a single time step equates to one trading day. Accordingly, a year is equivalent to *T*_*y*_ = 286 trading days, a month to *T*_*m*_ = 21 trading days, and a week to *T*_*w*_ = 5 trading days. Our analysis typically involves examining statistical properties derived from a series of *S* simulations. Furthermore, we incorporate transaction costs through brokerage fees *b* for each trade, an annual risk-free interest rate *R* for the agents’ risk-free assets, and an annual dividend yield *D* for the agents’ stock investments. These financial parameters, observed during the period from 2007 to 2018 for model calibration, are assumed to be constant for simplification, with the brokerage fees at *b* = 0.1%, the risk-free rate at *R* = 1%, and the dividend yield at *D* = 2%. These values are based on average figures from the London Stock Exchange, UK bond yields, and the FTSE 250 stock dividends, respectively. Each simulation cycle at time *t* comprises four primary steps, as outlined below:

i- *Initialization of Agent Parameters*: At the commencement of the simulation (*t* = 0), *I* agents are initialized with their individual parameters. Each agent, representing either an individual or institutional investor, manages a portfolio of stocks (equity) and risk-free assets (bonds) over time *t*. The specifics of these parameters are detailed in Section 8.2.2.
ii- *Establishment of Market Fundamentals*: Following the approach of other models, we set all market prices to *£*100 at the start (*P*^*j*^(*t* = 0)) and create *J* price time series $T^{j}\left(t\right)$ as jump processes, reflecting fundamental stock values. These values are derived from the enterprise value of companies, divided by the total number of outstanding stocks. This information is not fully accessible to the *I* agents. Instead, each agent *i* estimates the value $T^{j}\left(t\right)$ for stock *j* using their own cointegration rule $\kappa^{i,j}\left[T^{j}\left(t\right)\right]=B^{i,j}\left(t\right)$. The series $B^{i,j}\left(t\right)$ thus represent the perceived fundamental values of stock *j* over time *t* by agent *i*. We include in Fig 9 examples of such calculated enterprise values for various companies listed on the London Stock Exchange between 2006 and 2016. Additionally, Fig 10 illustrates the concept of cointegration by comparing the modelled fundamental values $T^{j}\left(t\right)$ with their approximations $B^{i,j}\left(t\right)$ by certain agents. From a set of *S* = 20 simulations, we calculate the average annual number of jumps in $T^{j}\left(t\right)$ to be 12.70 ± 1.85, the average jump amplitude $\left(T^{j}\left(t\right)-T^{j}\left(t-1\right)\right)/T^{j}\left(t\right)$ to be 5.90 ± 1.84%, and the average disparity between the biased and actual values $\left(T^{j}\left(t\right)-B^{i,j}\left(t\right)\right)/T^{j}\left(t\right)$ to be 2.37±1.36%. Agents employ these two sources of information—chartist and fundamental—for their stock pricing strategies.
iii- *Independent Forecasting and Trading by Agents*: In this model, each agent independently employs two reinforcement learning algorithms for market interaction. The specifics of these algorithms are elaborated in Section 8.2.3. The first algorithm, denoted as $F^{i}$, is tasked with devising the ideal econometric forecasting function. This function takes into account the unique aspects of the market’s microstructure and the agent’s own fundamental valuation, represented as $B^{i,j}\left(t\right)$. The forecast generated by $F^{i}$ is then fed into the second reinforcement learning algorithm, $T^{i}$. Algorithm $T^{i}$ is responsible for generating the optimal limit order for a double auction order book (as discussed in [86]) at that time step, integrating the forecast and various indicators related to market microstructure and the agent’s portfolio. An essential feature of this process is the filter function $G^{i}$, which determines the most advantageous time step for the agent to place a transaction order.
iv- *Populating and Processing the Order Book*: At each time step *t*, a set of *J* order books are populated with limit orders from agents for a particular stock *j*. These orders are organized such that buy orders are ranked in descending order of bid prices, and sell orders in ascending order of ask prices, each accompanied by the quantity of stocks offered for trade. The clearing of the order book occurs at this same time step *t*. It involves pairing buy and sell orders starting from the highest bid and lowest ask prices, progressing to the point where bid prices no longer exceed ask prices. The market price *P*^*j*^(*t* + 1) for stock *j* in the subsequent time step *t* is determined by the mid-price at this final matching point. Likewise, the trading volume *V*^*j*^(*t* + 1) is defined as the total quantity of stock *j* exchanged at time *t*. Additionally, the spread *W*^*j*^(*t* + 1) for stock *j* at time step *t* is calculated as the absolute difference between the mean of all bids and asks. It’s noteworthy that this spread, *W*^*j*^(*t*), is utilized in the agents’ stock pricing mechanism, rather than the conventional bid-ask spread, which is typically defined by the gap between the highest bid and the lowest ask.

**Fig 11:**
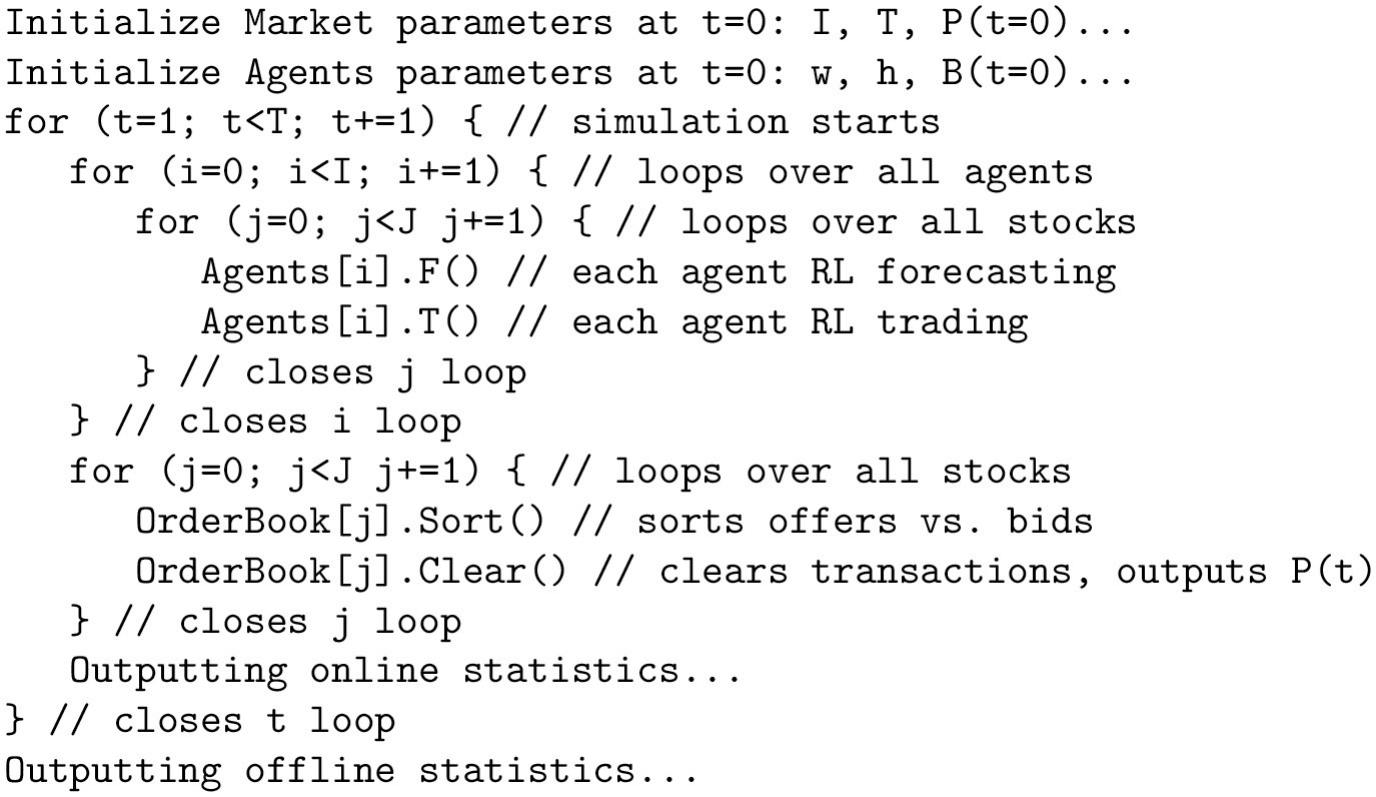
Pseudo-code of SYMBA’s iteration procedure.

The pseudo-code of SYMBA’s iteration procedure is found in Fig 11.

#### 8.2.2 Initialization parameters for agents

Our model integrates a variety of parameters at both individual agent and overall framework levels. As already stated in Section 3, the continuous and discrete uniform distributions are denoted by U(),U{}, while continuous and discrete normal distributions are represented by N(),N{}. At *step 1*, each agent *i* is assigned the following initial parameters:

- A trading window *w*^*i*^, determined by a uniform distribution $U\{T_{w},\tau^{i}\}$. This parameter influences the $G^{i}$ function, which calculates the optimal timing for purchasing stocks.

And as already mentioned in Section 3:

- An initial value of risk-free assets $A_{\text{bonds}}^{i}\left(t=0\right)$, following a normal distribution $N(0,10^{4})$. This represents the agent’s bonds or bank account balance, which increases when the agent shorts its stocks or longs equity.
- A quantity of stocks *Q*^*i*,*j*^(*t* = 0) for each stock *j*, derived from a discrete positive half-normal distribution $N^{+}\left\{0,100\right\}$. The total value of these stocks is given by $A_{\text{equity}}^{i}\left(t=0\right)=\sum_{j=0}^{J}Q^{i,j}\left(t=0\right)P^{j}\left(t=0\right)$, which the agent may decide to short sell in the market.
- An investment duration *τ*^*i*^, chosen from a uniform distribution $U\{T_{w},6T_{m}\}$. This parameter dictates the time frame after which the agent will liquidate its position, ranging from one week to six months in trading days.
- A memory span *h*^*i*^, obtained from a uniform distribution $U\{T_{w},T\}$. This interval represents the duration of historical data the agent considers for its learning process.
- A transaction gesture threshold *g*^*i*^, derived from a uniform distribution U(0.2,0.8). This parameter determines the agent’s willingness to transact at prices above or below its own stock valuation. The range of this parameter is influenced by the model’s gesture scalar *ζ*^*i*^, as discussed in Table 2 below.
- A reflexivity amplitude parameter *ρ*^*i*^, assigned from a uniform distribution U(0,100%). This parameter influences the agent’s approach to price valuation, balancing between technical market forecasts and fundamental pricing. It affects the first reinforcement learning algorithm’s action amplitude F.
- A reinforcement learning rate parameter *β*^*i*^, set from a uniform distribution U(0.05,0.20). This rate, applicable to both reinforcement algorithms $F^{i}$ and $T^{i}$, is based on findings from neuroscience literature [17, 18, 74].
- A drawdown threshold *l*^*i*^, defined as the year-to-date peak-to-bottom loss in net asset value. This parameter is drawn from a uniform distribution U(40%,50%). If the agent’s portfolio value drops below this threshold in any given time step *t*, the agent is considered bankrupt and is excluded from further market interactions. This threshold is higher than typical industry standards due to our model’s requirement for maintaining a constant number of agents, even in bankruptcy scenarios.

**Table 2:** Model hyperparameters and ranges for training: Lower bound (Low), upper bound (High), and increment step (Step).

Hyperparameter	Low	High	Step
Number of Agents (*I*)	500	5500	1000
Gesture Scalar (*ζ*^*i*^)	1.0	3.0	0.5
Fundamental Amplitude (*ν*)	0.1	1.5	0.2
Drawdown Threshold (L)	10	90	20

In the forthcoming Section 8.2.5, we explore various parameters within our model. Some of these parameters are fine-tuned as hyperparameters of the model, such as the drawdown limit *l*^*i*^, adjusted via a threshold $L\in N^{+}$, and the transaction gesture *g*^*i*^, modified through *ζ*^*i*^. Others derive from existing literature, like the reinforcement learning rate *β*^*i*^. Parameters like the reflexivity amplitude parameter *ρ*^*i*^ are incorporated as learned variables in the agent’s reinforcement learning process. Additionally, certain parameters are preset, including the values of agents’ bond portfolios $A_{\text{bonds}}^{i}$, equity portfolios $A_{\text{equity}}^{i}$, the investment horizon *τ*^*i*^, and time intervals *w*^*i*^ and *h*^*i*^. These elements contribute to the foundational structure of our model’s architecture.

#### 8.2.3 Agent reinforcement learning: Initial algorithm

Here, we delve into *step 3* above, highlighting the two core reinforcement learning algorithms: $F^{i}$, responsible for accurate price prediction, and $T^{i}$, focused on effective trading based on those predictions. As already said in Section 3, each agent *i* runs these algorithms independently, applied to each stock *j* at every time step *t*. The agents employ a direct policy search approach, where the probability of each action is determined directly from the policy, bypassing any action-value function as in the Generalized Policy Iteration theorem [87]. The action-state pairs for these algorithms are 729 and 972 respectively. We establish the states S, actions A, and returns R for both algorithms as follows.

The first algorithm, $F^{i}$, allows the agent to track long-term stock price volatility ($s_{0}^{F}$), short-term volatility ($s_{1}^{F}$), and the difference between its fundamental valuation and the current market price ($s_{2}^{F}$). Based on this information, the agent optimizes its price forecasting over its investment horizon *τ*^*i*^ by testing three actions through direct policy search: adopting a basic econometric forecasting method focused on mean-reverting, averaging, or trend-following ($a_{0}^{F}$), selecting the duration of the past interval for forecasting ($a_{1}^{F}$), and determining the influence of its own fundamental stock pricing in the combined future price estimate, which includes both fundamentalist and chartist perspectives ($a_{2}^{F}$).

*States $S^{F}$*: The algorithm $F^{i}$ operates within a state space $S^{F}=\left\{s_{0}^{F},s_{1}^{F},s_{2}^{F}\right\}$, comprising 27 dimensions. Each state component can take on values of 0, 1, or 2. Agents calculate the variances $\sigma_{L}^{2}$ and $\sigma_{S}^{2}$ of stock prices *P*^*j*^(*t*) over specific time frames.

-
$\sigma_{L}^{2}$ , representing long-term volatility, is evaluated and stored in a time series, sorted ascendingly and truncated to match the agent’s memory span *h*^*i*^. Its percentile ranking at time *t* determines $s_{0}^{F}$, classifying it into three categories: the percentile of its present value at time step *t* sets $s_{0}^{F}=0$ if it is below 25%, $s_{0}^{F}=2$ if it is above 75%, and $s_{0}^{F}=1$ otherwise.
- Similarly, $\sigma_{S}^{2}$, indicating short-term volatility, is processed and categorized in the same way, providing insight into the short-term market dynamics of stock *j*.
- The discrepancy between the market price and the agent’s fundamental valuation is measured by averaging the relative difference $\left|P^{j}\left(t\right)-B^{i,j}\left(t\right)\right|/P^{j}\left(t\right)$ over a set interval [*t* − 3*τ*^*i*^, *t*], and sets $s_{2}^{F}=0$ if it is below 10%, $s_{2}^{F}=2$ if it is above 30%, and $s_{2}^{F}=1$ otherwise.

*Actions $A^{F}$*: In the context of the reinforcement learning framework $F^{i}$, we consider an action $a^{F}$ that is part of a set $A^{F}=\left\{a_{0}^{F},a_{1}^{F},a_{2}^{F}\right\}$, which spans 27 possible states. Here, each action $a_{0}^{F},a_{1}^{F},a_{2}^{F}$ can independently assume one of the values 0, 1, or 2. The selection of these actions is governed by a direct policy search, as detailed further, and depends on whether the agent is in a state of exploration or exploitation. Initially, each agent calculates two separate mean values $\langle P_{\left[t-2T,t-T\right]}^{j}\left(t\right)\rangle$ and $\langle P_{\left[t-T,t\right]}^{j}\left(t\right)\rangle$ of historical stock prices, where *T* is defined as $T=\left(1+a_{1}^{F}\right)\tau^{i}/2$. Subsequently, the econometric mechanism calculates:

$$\begin{array}{c} {\hat{P}}^{i,j}\left(t\right)=P^{j}\left(t\right)+\left\langle P_{\left[t-2T,t-T\right]}^{j}\left(t\right)\right\rangle -\left\langle P_{\left[t-T,t\right]}^{j}\left(t\right)\right\rangle \end{array}$$ (3)

$$\begin{array}{c} {\hat{P}}^{i,j}\left(t\right)=\frac{1}{2}\left\langle P_{\left[t-2T,t-T\right]}^{j}\left(t\right)\right\rangle +\frac{1}{2}\left\langle P_{\left[t-T,t\right]}^{j}\left(t\right)\right\rangle \end{array}$$ (4)

$$\begin{array}{c} {\hat{P}}^{i,j}\left(t\right)=P^{j}\left(t\right)-\left\langle P_{\left[t-2T,t-T\right]}^{j}\left(t\right)\right\rangle +\left\langle P_{\left[t-T,t\right]}^{j}\left(t\right)\right\rangle \end{array}$$ (5)

applicable for $a_{0}^{F}=0,1,2$ respectively. These correspond to strategies of mean-reversion, use of moving averages, and trend tracking. Therefore, actions $a_{0}^{F}$ and $a_{1}^{F}$ are related to technical analysis, with $a_{0}^{F}$ dictating the choice of econometric forecasting approach and $a_{1}^{F}$ defining the interval length for these forecasts. The third action, $a_{2}^{F}$, influences the blend of the selected technical forecast ${\hat{P}}^{i,j}\left(t\right)$ with the agent’s fundamental valuation $B^{i,j}\left(t\right)$, generating the agent’s projection:

$$\begin{array}{c} H^{i,j}\left(t\right)=\alpha {\hat{P}}^{i,j}\left(t\right)+\left(1-\alpha \right)B^{i,j}\left(t\right) \end{array}$$ (6)

where α∈R is chosen based on the agent’s reflexivity *ρ*^*i*^. If *ρ*^*i*^ ≤ 50%, then *α* is set to 0, *ρ*^*i*^, 2*ρ*^*i*^ for $a_{2}^{F}=0,1,2$ respectively. Conversely, if *ρ*^*i*^ > 50%, *α* takes the values 2*ρ*^*i*^ − 1, *ρ*^*i*^, 1 for $a_{2}^{F}=0,1,2$. Thus, with $a_{2}^{F}=2$, the agent adjusts the weight assigned to its chartist versus fundamentalist valuation methods.

*Returns $R^{F}$*: The reinforcement learning scheme $F^{i}$ then determines the percentage discrepancy between the agent’s prior stock price forecast *H*^*i*,*j*^(*t* − *τ*^*i*^) made *τ*^*i*^ time steps earlier, and the current actual price *P*^*j*^(*t*):

$$\begin{array}{c} \frac{\left|H^{i,j}\left(t-\tau^{i}\right)-P^{j}\left(t\right)\right|}{P^{j}\left(t\right)} \end{array}$$ (7)

This value is recorded at each time step, organized in ascending order, and maintained at a length matching the memory interval *h*^*i*^ of the agent. The percentile rank of this value at time step *t* is used to assign a discrete return value $r^{F}$ from the set $R^{F}=\left\{4,2,1,-1,-2,-4\right\}$, corresponding to the intervals [0%, 5%(, [5%, 25%(, [25%, 50%(, [50%, 75%(, [75%, 95%(, [95%, 100%] respectively.

*Policy $\pi^{F}$*: The reinforcement learning mechanism periodically refines its policy $\pi_{t}^{F}\left(s_{t-\tau^{i}}^{F},a_{t-\tau^{i}}^{F}\right)$ at each interval *t*. This refinement is influenced by the agent’s learning rate, denoted by *β*. The following equations are employed, iteratively run $\left|r^{F}\right|$ times, to enhance the likelihood of selecting an optimal action $a^{F⋆}$ in the given state $s^{F}$. The process aims to increment the policy’s probability for this optimal action relative to other possible actions, $\forall a^{F}\neq a^{F⋆}$:

$$\begin{array}{c} \pi_{t+1}^{F}\left(s^{F},a^{F⋆}\right)=\pi_{t}^{F}\left(s^{F},a^{F⋆}\right)+\beta [1-\pi_{t}^{F}\left(s^{F},a^{F⋆}\right)] \end{array}$$ (8)

$$\begin{array}{c} \pi_{t+1}^{F}\left(s^{F},a^{F}\right)=\pi_{t}^{F}\left(s^{F},a^{F}\right)-\beta \pi_{t}^{F}\left(s^{F},a^{F}\right) \end{array}$$ (9)

Moreover, the algorithm incorporates an off-policy approach at intervals of *τ*^*i*^/*T*_*m*_ + 2. This method calculates the action that should have ideally been taken by $F^{i}$ *τ*^*i*^ steps prior, now informed by current price and forecast accuracy. Subsequently, it updates the policy $\pi^{F}$ using the agent’s learning rate *β*, applied $|r^{F}|=4$ times, to recognize and adjust for the action now identified as optimal.

#### 8.2.4 Implementation of the secondary agent-based reinforcement learning algorithm

This secondary approach enables the agent to dynamically assess the progression of stock prices, as initially determined by the primary algorithm ($s_{0}^{T}$). It also evaluates market volatility ($s_{1}^{T}$), the status of risk-averse assets ($s_{2}^{T}$), the current amount of stocks held ($s_{3}^{T}$), and volume of trades executed ($s_{4}^{T}$). Using this gathered information, the agent refines its investment strategies. It does so by employing a direct policy search method, where it decides whether to hold, buy, or sell stocks in particular quantities ($a_{0}^{T}$), and determines the transaction price in response to market supply and demand dynamics ($a_{1}^{T}$).

*States $S^{T}$*: The agent’s decision-making process in the algorithm $T^{i}$ relies on a state $s^{T}$ within the set $S^{T}=\left\{s_{0}^{T},s_{1}^{T},s_{2}^{T},s_{3}^{T},s_{4}^{T}\right\}$. This set encompasses a 108-dimensional space, where $s_{0}^{T}$, $s_{1}^{T}$, and $s_{4}^{T}$ can take on values from the set {0, 1, 2}, and $s_{2}^{T}$, $s_{3}^{T}$ from {0, 1}.

- The agent calculates the ratio *μ* = (*H*^*i*,*j*^(*t*) − *P*^*j*^(*t*))/*P*^*j*^(*t*) and logs it in either ***μ***_−_ or ***μ***_+_ time series, based on its being negative or positive, respectively. These series are sorted in ascending order and capped to match the agent’s memory span *h*^*i*^. The agent’s current percentile value ${\bar{\mu }}_{-}$ in ***μ***_−_ at time *t* assigns $s_{0}^{T}=0$ if it’s under 95%, and $s_{0}^{T}=1$ if not. Likewise, ${\bar{\mu }}_{+}$ in ***μ***_+_ determines $s_{0}^{T}$ to be 1 if under 5%, and $s_{0}^{T}=2$ otherwise. The state $s_{0}^{T}$ thus reflects the econometric prediction *μ* from the prior algorithm $F^{i}$, indicating a decrease, stability, or increase in stock *j* prices in future *τ*^*i*^ time steps.
- The agent documents the previously computed variance $\sigma_{L}^{2}$ of stock prices *P*^*j*^(*t*) in a time series for the interval [*t* − 3*τ*^*i*^, *t*]. This series is sorted and truncated to align with the agent’s memory span *h*^*i*^. The agent’s current percentile value at time *t* sets $s_{1}^{T}=0$ if below 33%, $s_{1}^{T}=2$ if above 67%, and $s_{1}^{T}=1$ in other cases, thus guiding the agent in understanding longer-term stock price volatility.
- The agent assigns $s_{2}^{T}=0$ if its risk-free asset value $A_{\text{bonds}}^{i}\left(t\right)$ falls below 60% of its initial value $A_{\text{bonds}}^{i}\left(t=0\right)$, and $s_{2}^{T}=1$ otherwise. This assists the agent in monitoring its risk-free asset size for adopting suitable investment strategies.
- The agent sets $s_{3}^{T}=0$ if the current value of its stock holdings $A_{\text{equity}}^{i}\left(t\right)$ is less than 60% of the starting value $A_{\text{equity}}^{i}\left(t=0\right)$, and $s_{3}^{T}=1$ otherwise. This process aids the agent in tracking the value of its stock holdings for strategic decision-making.
- The agent logs the trading volumes *V*^*j*^(*t*) at each time step in a series, sorted in ascending order and truncated as per the agent’s memory period *h*^*i*^. The current percentile value at time *t* determines $s_{4}^{T}=0$ if *V*^*j*^(*t*) = 0, $s_{4}^{T}=1$ if below 33%, and $s_{4}^{T}=2$ in other cases. This informs the agent about market activity levels, aiding in setting appropriate bid or ask prices for transactions.

*Actions $A^{T}$*: In the context of the reinforcement learning model $T^{i}$, we introduce a set of actions, denoted by $A^{T}=\left\{a_{0}^{T},a_{1}^{T}\right\}$, where each action $a^{T}$ is a part of this set. This action set is characterized by a dimensionality of 9. Actions $a_{0}^{T}$ and $a_{1}^{T}$ are capable of adopting discrete values from the set {0, 1, 2}, determined via a process of direct policy search (referenced in the subsequent section). Action $a_{0}^{T}$ is twofold in its representation: it signifies both the amount of stocks and the type of transaction order (sell, hold, or buy) that the agent decides to place in the order book. In this framework, each agent adheres to a long-only trading strategy, involving the purchase of stocks at a specific price, holding them for a predetermined duration, and ultimately selling them, ideally at a higher price. The role of action $a_{1}^{T}$ is to indicate the agent’s willingness to be flexible about the trading price. These actions are contingent upon the agent’s assessment of stock *j*’s price, as evaluated through the initial algorithm $F^{i}$. The agent’s bid price $P_{\text{bid}}^{i,j}\left(t\right)$ is formulated as follows:

$$\begin{array}{c} P_{\text{bid}}^{i,j}\left(t\right)=\text{min}\left[H^{i,j}\left(t\right),P^{j}\left(t\right)\right]+g^{i}W^{j}\left(t-1\right) \end{array}$$ (10)

$$\begin{array}{c} P_{\text{bid}}^{i,j}\left(t\right)=\text{min}\left[H^{i,j}\left(t\right),P^{j}\left(t\right)\right] \end{array}$$ (11)

$$\begin{array}{c} P_{\text{bid}}^{i,j}\left(t\right)=\text{min}\left[H^{i,j}\left(t\right),P^{j}\left(t\right)\right]-g^{i}W^{j}\left(t-1\right) \end{array}$$ (12)

corresponding to $a_{1}^{T}$ values of 0, 1, 2, respectively. It’s important to note that *g*^*i*^ represents the agent’s trading gesture and *W*^*j*^(*t* − 1) denotes the market spread of stock *j* at the previous time step. Thus, the term ±*g*^*i*^*W*^*j*^(*t* − 1) reflects the agent’s more lenient or stringent approach to the trading conditions, influenced by market factors such as *W*^*j*^(*t* − 1) and the trading volumes represented by $s_{4}^{T}$. The agent’s ask price $P_{\text{ask}}^{i,j}\left(t\right)$ is established as:

$$\begin{array}{c} P_{\text{ask}}^{i,j}\left(t\right)=\text{max}\left[H^{i,j}\left(t\right),P^{j}\left(t\right)\right]-g^{i}W^{j}\left(t-1\right) \end{array}$$ (13)

$$\begin{array}{c} P_{\text{ask}}^{i,j}\left(t\right)=\text{max}\left[H^{i,j}\left(t\right),P^{j}\left(t\right)\right] \end{array}$$ (14)

$$\begin{array}{c} P_{\text{ask}}^{i,j}\left(t\right)=\text{max}\left[H^{i,j}\left(t\right),P^{j}\left(t\right)\right]+g^{i}W^{j}\left(t-1\right) \end{array}$$ (15)

Consider the case when $a_{1}^{T}$ takes on the values of {0, 1, 2}. In this context, *Q*^*i*,*j*^(*t*) represents the number of shares of stock *j* held by investor *i* at time *t*. Here, the action $a_{0}^{T}=0$ is equivalent to investor *i* placing a sell order for their entire holding of stock *j* at the asking price $P_{\text{ask}}^{i,j}\left(t\right)$. Conversely, $a_{0}^{T}=1$ implies that investor *i* does not make any transaction, maintaining their current position. Lastly, $a_{0}^{T}=2$ denotes a buy order, where the investor purchases an amount of stock *j* determined by the formula $A_{\text{bonds}}^{i}\left(t\right)/\left[P_{\text{ask}}^{i,j}\left(t\right)J\right]$, at the bid price $P_{\text{bid}}^{i,j}\left(t\right)$. It is important to note that in this formula, *J* is included in the denominator to facilitate effective management of a diversified portfolio.

*Filter function $G^{i}$*: The decision of investor *i* to send an order for stock *j* at time step *t* to the order book is governed by the output of the function $G^{i}$. This function is designed to delay the sending of a transaction order until the most advantageous time step. To achieve this, $G^{i}$ maintains a time series, recording at each time step the maximum value of the action-value function $\text{arg}\;{\text{max}}_{a}Qt\left(s,a\right)$, which is then organized in ascending order. The decision to execute a trade is based on comparing the current percentile $p_{Q}\left(t\right)$ of this series with the ratio of time elapsed since the last transaction *k*^*i*,*j*^(*t*) to the investor’s individual trading window *w*^*i*^. An order is sent to the order book only if the condition $p_{Q}\left(t\right)<k^{i,j}\left(t\right)/w^{i}$ is satisfied. Note that while $G^{i}$ filters the initiation of trades, it does not apply to exit strategies, which are executed at the investor’s predetermined investment horizon *τ*^*i*^.

*Returns $R^{T}$*: The algorithm $T^{i}$ calculates the difference in cash flow between the current net asset value of investor *i*’s portfolio and its value had the previous actions taken *τ*^*i*^ time steps earlier not occurred. This is given by:

$$\begin{array}{c} Q_{\text{OB}}^{i,j}\left(t-\tau^{i}\right)\left[P^{j}\left(t\right)-P_{\text{OB}}^{i,j}\left(t-\tau^{i}\right)\right] \end{array}$$ (16)

In this equation, $Q_{\text{OB}}^{i,j}\left(t-\tau^{i}\right)$ and $P_{\text{OB}}^{i,j}\left(t-\tau^{i}\right)$ represent the quantity and price of stock *j* cleared in the order book at time *t* − *τ*^*i*^ for investor *i* and their trading counterpart. These values might differ from those initially sent by investor *i*, due to partial order fulfillment and the setting of the transaction price by the order book at the mid-price with the counterparty’s order price. These values are logged in a time series at each time step, sorted in ascending order, and truncated to maintain a length corresponding to the memory interval *h*^*i*^ of the investor. The percentile of this value at time *t* determines the discrete return value $r^{T}$ in the set $R^{T}=4,2,1,-1,-2,-4$, corresponding to the intervals [0%, 5%, [5%, 25%(, [25%, 50%(, [50%, 75%(, [75%, 95%(, [95%, 100%].

*Policy $\pi^{T}$*: In the final step, the reinforcement learning algorithm adjusts its policy $\pi_{t}^{T}\left(s_{t-\tau^{i}}^{T},a_{t-\tau^{i}}^{T}\right)$ after every *τ*^*i*^ time steps following each transaction carried out by the agent. This adjustment is made based on the agent’s learning rate *β*, and the following equations are iterated $\left|r^{T}\right|$ times. The aim is to prioritize an action, denoted as $a^{T⋆}$, in state $s^{T}$ by increasing the policy probability associated with this action over other actions, denoted as $\forall a^{T}\neq a^{T⋆}$:

$$\begin{array}{c} \pi_{t+1}^{T}\left(s^{T},a^{T⋆}\right)=\pi_{t}^{T}\left(s^{T},a^{T⋆}\right)+\beta \left[1-\pi_{t}^{T}\left(s^{T},a^{T⋆}\right)\right] \end{array}$$ (17)

$$\begin{array}{c} \pi_{t+1}^{T}\left(s^{T},a^{T}\right)=\pi_{t}^{T}\left(s^{T},a^{T}\right)-\beta \pi_{t}^{T}\left(s^{T},a^{T}\right) \end{array}$$ (18)

Additionally, the algorithm employs an off-policy method every *τ*^*i*^/*T*_*m*_ + 2 time steps. This method calculates the optimal action that $T^{i}$ should have taken *τ*^*i*^ time steps ago, considering the realized price and forecast accuracy, and updates the policy $\pi^{T}$ using the agent’s learning rate *β*. This update process is repeated $|r^{T}|=4$ times since the associated action is considered optimal.

Furthermore, it is important to note that both algorithms F and T utilize discretized and handcrafted action-state spaces. This choice is motivated by the need to conserve computational resources and address a key limitation in applying Multi-Agent Systems (MAS) to financial research, which is the requirement for substantial computational power. Furthermore, the general intuition behind our definition of such state and action spaces has been the *Fundamental Theorem of Asset Pricing* [88], where present asset prices are estimated from time-discounted future prices expectations. In a similar vein, our reinforcement learning framework for the agent is structured around a forecasting component $F^{i}$ and a trading component $T^{i}$, a design approach reminiscent of recent models such as [49] (see Section 8.2.3 for more details).

#### 8.2.5 Alignment with real data

*Model assumptions*. The SYMBA model is based on two fundamental assumptions: i) that the behavior of the simulated agents accurately mirrors that of real-world investors, and ii) that the transaction limit orders simulated in the order book faithfully represent the dynamics and characteristics of actual stock market orders. Regarding the former, our approach simplifies the interaction of any agent, regardless of its behavior or strategy, into three distinct possibilities: buying, selling, or holding stocks (a long-only strategy). Concerning the latter, it is worth noting that the dynamics of order books have been extensively documented in the literature [89], allowing for a rigorous design.

*Model limitations*. In addition to these core hypotheses, we also acknowledge several limitations and consistency issues inherent to all financial Multi-Agent Systems (MAS): i) reliance on the generation of virtual fundamentals $T^{j}\left(t\right)$, ii) absence of portfolio diversification across different asset classes, iii) lack of various trading strategies (e.g., short-selling, leveraging, derivatives, metaorders, market orders, etc.), iv) omission of intraday and seasonal market effects, and v) absence of legal and regulatory constraints. While some of these limitations may appear challenging, their impact and significance are inherent in nearly all other econometric and modeling approaches within quantitative finance. Additionally, modeling market activity through a market microstructure derived from a centralized order book that processes transaction orders from multiple trading agents aligns closely with real stock markets, making it empirically relevant.

*Training and testing data*. We fine-tuned the MAS stock market simulator using real stock market data. The computations were performed on a Mac Pro equipped with a 3.5 GHz 6-Core Intel Xeon E5 processor and 16 GB of 1866 MHz DDR memory. To accomplish this, we utilized high-quality, industry-grade daily closing prices and trading volumes for a total of 4,313 stocks listed on the London Stock Exchange (LSE), covering the period from January 15th, 2007, to January 19th, 2018. As said in Section 3, these quotes include the date, opening price, highest price, lowest price, closing price, and trading volume for each day. Importantly, these figures are directly sourced from the London Stock Exchange (LSE) and are not a compilation of data from smaller exchanges (i.e. consolidated data). For our market microstructure analysis, we applied the following data filtering steps: i) removal of stock-split effects, and ii) inclusion of only those stocks that were continuously traded throughout this time frame. As a result of this data curation, our initial stock universe was reduced to 640 stocks. We calibrated the MAS hyperparameters using a random sample of half of these stocks as a training set. Remarkably, we observed a high degree of statistical stability in the training set compared to the other half. We attribute this stability to the unique characteristics of stock market data, particularly the absence of market arbitrage, which is closely related to the stylized facts previously mentioned.

*Optimization of hyperparameters*. The hyperparameters subject to calibration include the number of agents (*I*), the agent transaction gesture factor ($\zeta^{i}\in N$, which scales the gesture parameter *g*^*i*^ initialized for each agent at *t* = 0), the parameter governing the generation of fundamental values (*ν*, representing the amplitude of the fundamental time series $T^{j}$), and the drawdown threshold (the upper limit of the drawdown, initialized at *t* = 0 for each agent). We evaluated various combinations of hyperparameters against the training dataset, and the details are presented in Table 2. The optimization process involved a total of 1200 simulations, each comprising 20 runs for statistical reliability.

*Sensitivity analysis*. During the optimization process, we conducted a sensitivity analysis to assess how the model responds to different hyperparameter ranges. This analysis aimed to identify regions of non-linearity in terms of calibration against real data. Specifically, we observed that increasing the number of agents (*I*) had a linear effect on reducing short-term price volatilities. Larger values of gesture scalar (*ζ*^*i*^) and fundamental amplitude (*ν*) led to a linear increase in absolute daily price returns. Notably, the model exhibited minimal sensitivity to large drawdown thresholds (L>30%), as these values had a limited impact on agent survivability rates.

*Dataset variability*. One potential concern regarding the significance of calibrating SYMBA for replicating real financial market behaviors is the dependence of the calibration process on the specific market (e.g., different stock exchanges) and the chosen trading periods (e.g., market conditions and financial crises). To gain insight into the performance of SYMBA, we focus on the London Stock Exchange (LSE) as a case study.

**Fig 12:**
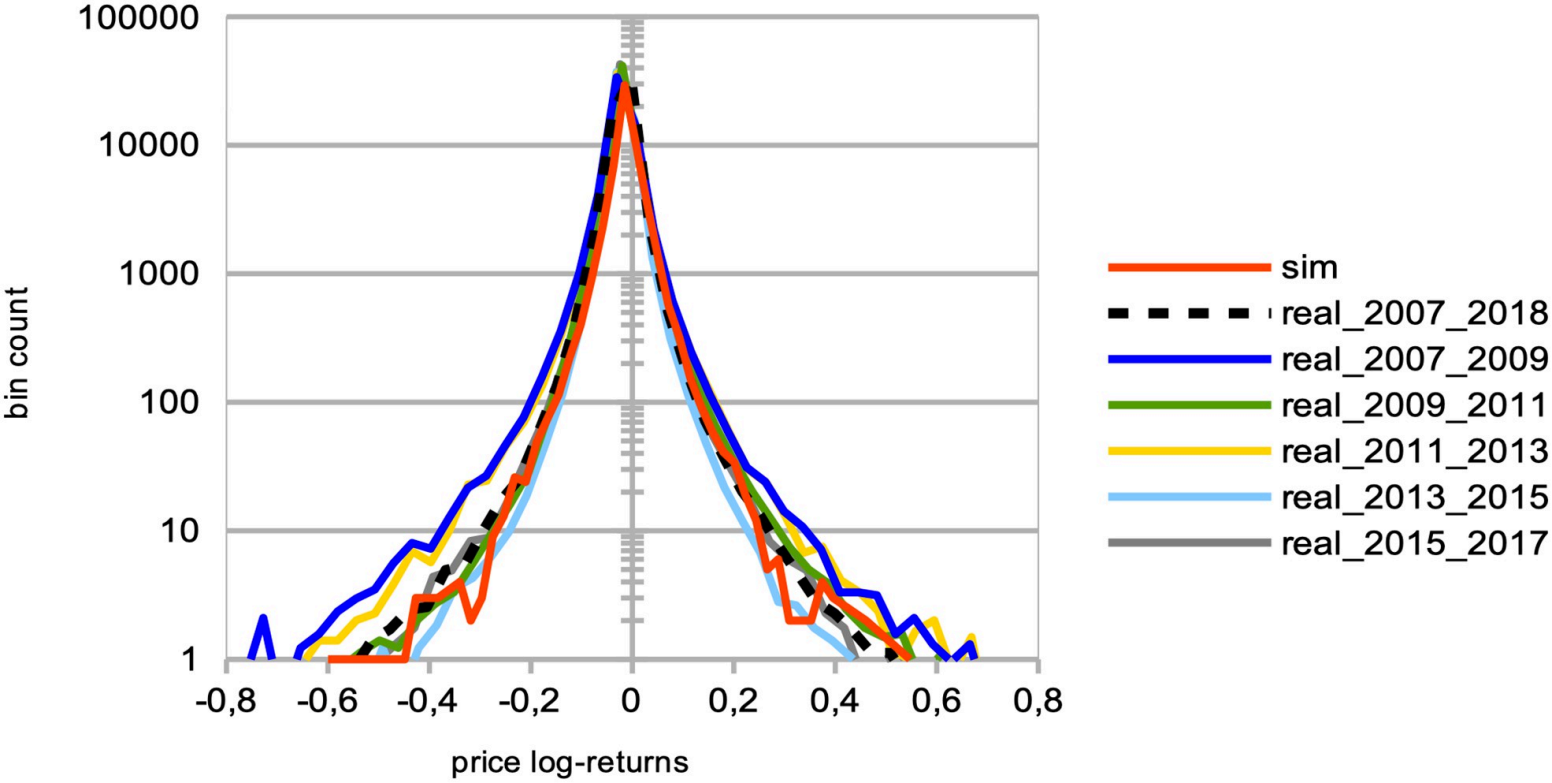
Distributions of the price log-returns for simulation data vs. LSE data for different time intervals. Comparative Distribution of Logarithmic Price Returns: The dashed black curve represents real data from the LSE between years 2007 and 2018, while the continuous red curve represents simulated data. These simulations were generated using parameters *I* = 500, *J* = 1, *T* = 2875, and *S* = 20. This is compared with two-years batches of real data from the LSE, between years 2007 and 2009 (darker blue curve), 2009 and 2011 (green curve), 2011 and 2013 (yellow curve), 2013 and 2015 (lighter blue curve), and 2015 and 2017 (grey curve). Notice the bin count of each curve is normalized for better comparison.

Fig 12 presents the distributions of logarithmic price returns for SYMBA (depicted by the red curve) in comparison to those of the LSE across various time intervals. These intervals span from 2007 to 2018 (shown as a dashed black curve), 2007 to 2009 (depicted by the darker blue curve), 2009 to 2011 (illustrated by the green curve), 2011 to 2013 (represented by the yellow curve), 2013 to 2015 (displayed as the lighter blue curve), and 2015 to 2017 (shown as the grey curve). It is important to note that: i- the bin count for each curve has been normalized to facilitate a more effective comparison; ii- only the stocks that were continuously traded during these respective time spans were included for the statistics (cf. survivorship bias mentioned at the beginning of Section 3).

Remarkably, the observed variations among these curves are relatively modest, with minor fluctuations evident during specific periods. Notably, a slight shift towards negative skewness is observable in the curves corresponding with the 2007–2008 Global Financial Crisis and the 2012 European Debt Crisis.

This analysis demonstrates that, despite variations in market conditions and crises, SYMBA’s performance in emulating the LSE’s price return distributions remains consistent. These findings lend support to the robustness and applicability of SYMBA in modeling real financial market dynamics.

**Fig 13:**
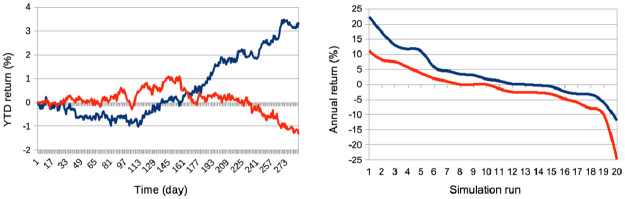
Comparison of SYMBA agents vs. random trading agents’ performance. At the conclusion of 90% of the total simulation time, we aim to contrast the top 10% performers among our multi-agent system (MAS) stock market simulator (represented by blue curves) with the top 10% performers in a market simulated with randomly trading noise agents (illustrated by red curves). To achieve this, we evaluate their performance during the remaining 10% of our overall simulation duration, utilizing averaged equity curves as their year-to-date returns across 20 simulations (left), and the averaged, sorted annual returns from each of these 20 simulations (right). These simulations are generated using the following parameters: *I* = 500, *J* = 1, and *T* = 2875.

*Model comparison*. Existing literature, such as [85], extensively discusses the substitution of markets for individual rationality, exploring whether markets eliminate irrational individuals or whether individuals adapt and learn market rules. Fig 13 presents agent learning curves that can be utilized for model comparison, particularly when compared to recent order book models combined with reinforcement learning [49] and the earlier generation of MAS featuring zero-intelligence agents [85] as baseline references.

#### 8.2.6 Calibration statistics

**Fig 14:**
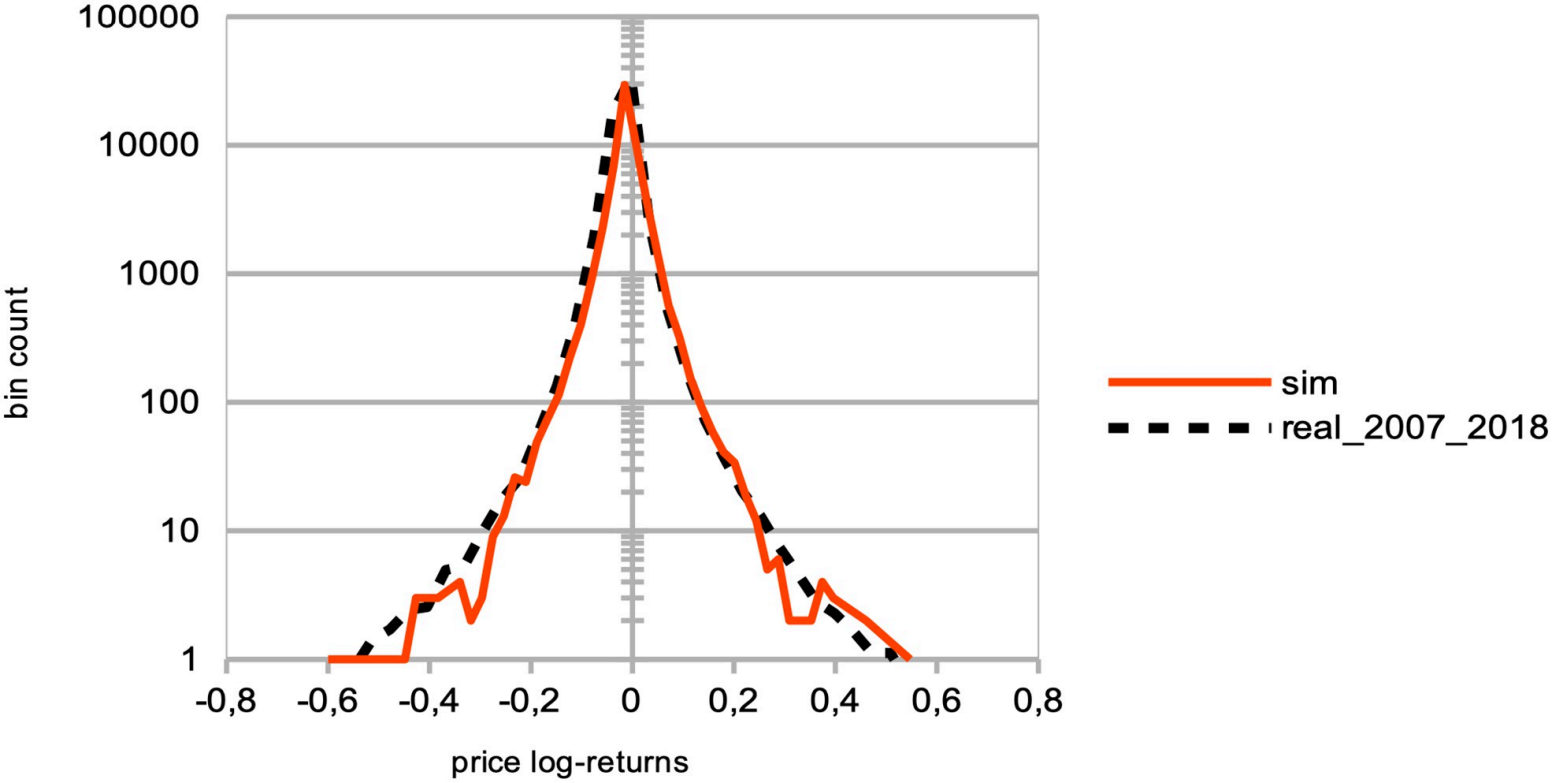
Comparative distribution of logarithmic price returns. The dashed black curve represents real data, while the continuous red curve represents simulated data. These simulations were generated using parameters *I* = 500, *J* = 1, *T* = 2875, and *S* = 20.

**Fig 15:**
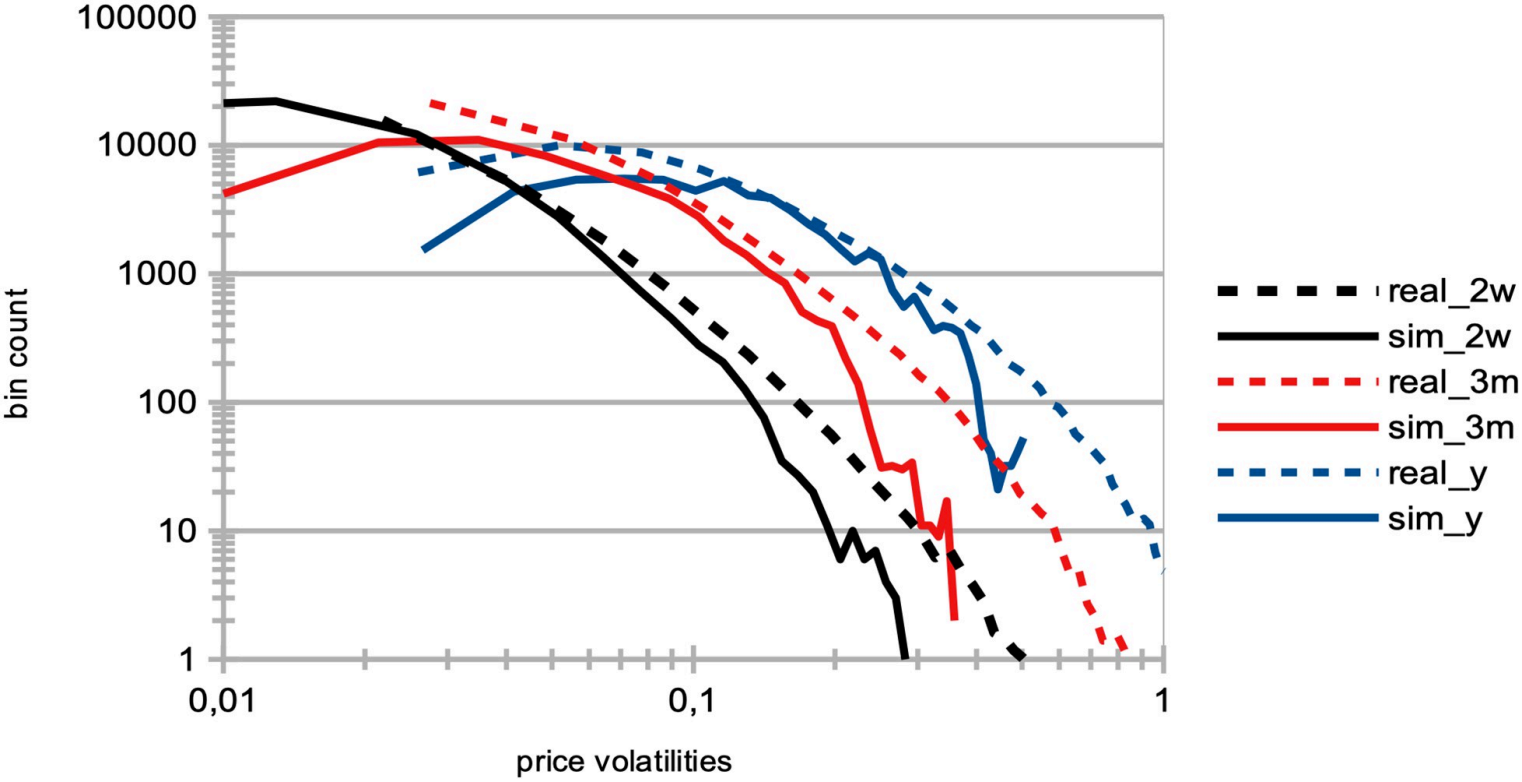
Volatility distribution for different time intervals. This figure illustrates the distribution of volatilities, computed at two weeks (black), three months (red), and one year (blue) intervals for both real (dashed curves) and simulated (continuous curves) data. The simulations were generated using parameters *I* = 500, *J* = 1, *T* = 2875, and *S* = 20.

**Fig 16:**
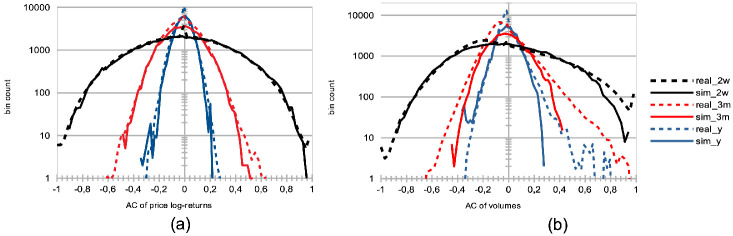
Autocorrelation distributions of the log-price returns and volumes for different time intervals. Figures (a) and (b) depict the autocorrelation distributions for (a) logarithmic price returns and (b) trading volumes, respectively, at each time step *t* across intervals [*t* − Δ, *t*] and [*t* − 2Δ, *t* − Δ]. Lags Δ of two weeks (black), three months (red), and one year (blue) are examined for both real (dashed curves) and simulated (continuous curves) data. Simulations had parameters *I* = 500, *J* = 1, *T* = 2875, and *S* = 20.

**Fig 17:**
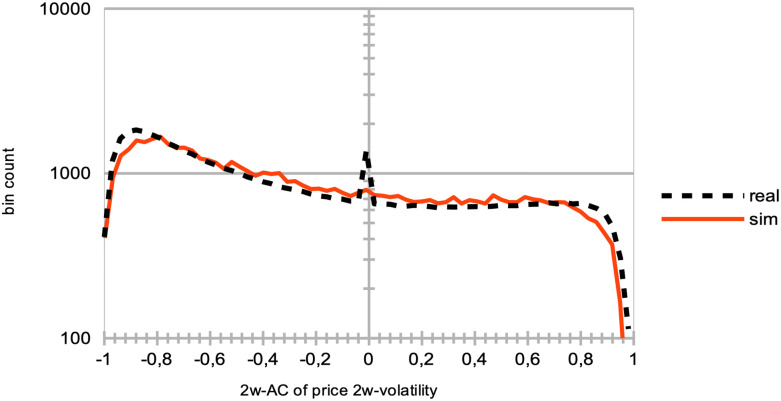
Autocorrelation distribution of two weeks-interval volatilities. This figure illustrates the distribution of autocorrelations of two weeks-interval volatilities at each time step *t* between intervals [*t* − Δ, *t*] and [*t* − 2Δ, *t* − Δ] for Δ = 2*T*_*w*_, for both real (dashed black curve) and simulated (continuous red curve) data. The simulations were generated using parameters *I* = 500, *J* = 1, *T* = 2875, and *S* = 20.

**Fig 18:**
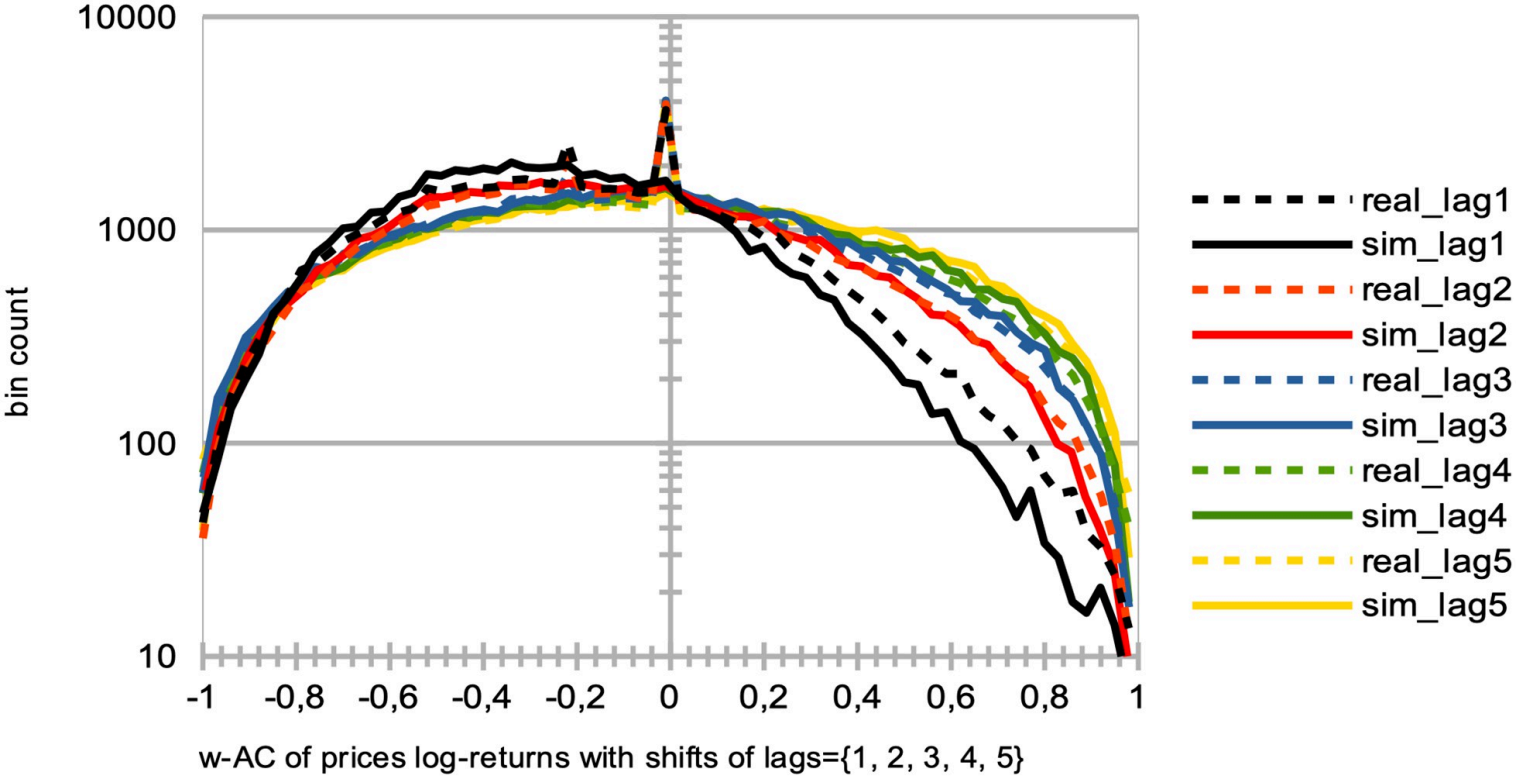
Autocorrelation distributions of log-price returns with various time-shift intervals. This figure shows the means of autocorrelations of logarithmic returns of prices at each time step *t* between intervals [*t* − *T*_*w*_, *t*] and [*t* − *T*_*w*_ − *δ*, *t* − *δ*], for shifts *δ* of one day (black), two days (red), three days (blue), four days (green), and five days (yellow). These calculations are based on both real (dashed curves) and simulated (continuous curves) data. The simulations were generated using parameters *I* = 500, *J* = 1, *T* = 2875, and *S* = 20.

**Fig 19:**
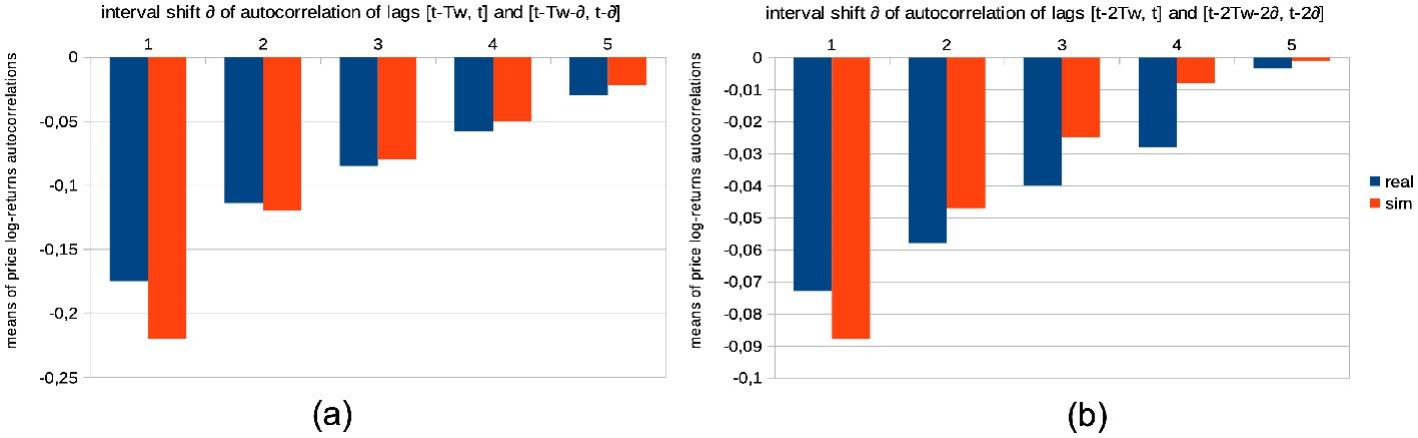
Mean autocorrelations of log-price returns for various time-shifts intervals. (a) Autocorrelation Means of Logarithmic Price Returns for Shifts *∂* ∈ {1, 2, 3, 4, 5}: These figures depict autocorrelation means of logarithmic returns at time t over intervals [*t* − *T*_*w*_, *t*] and [*t* − *T*_*w*_ − *∂*, *t* − *∂*]. (b) Shows similar means for intervals [*t* − 2*T*_*w*_, *t*] and [*t* − 2*T*_*w*_ − 2*∂*, *t* − 2*∂*]. Both plots feature real (blue) and simulated (red) data, using parameters *I* = 500, *J* = 1, *T* = 2875, and *S* = 20.

**Fig 20:**
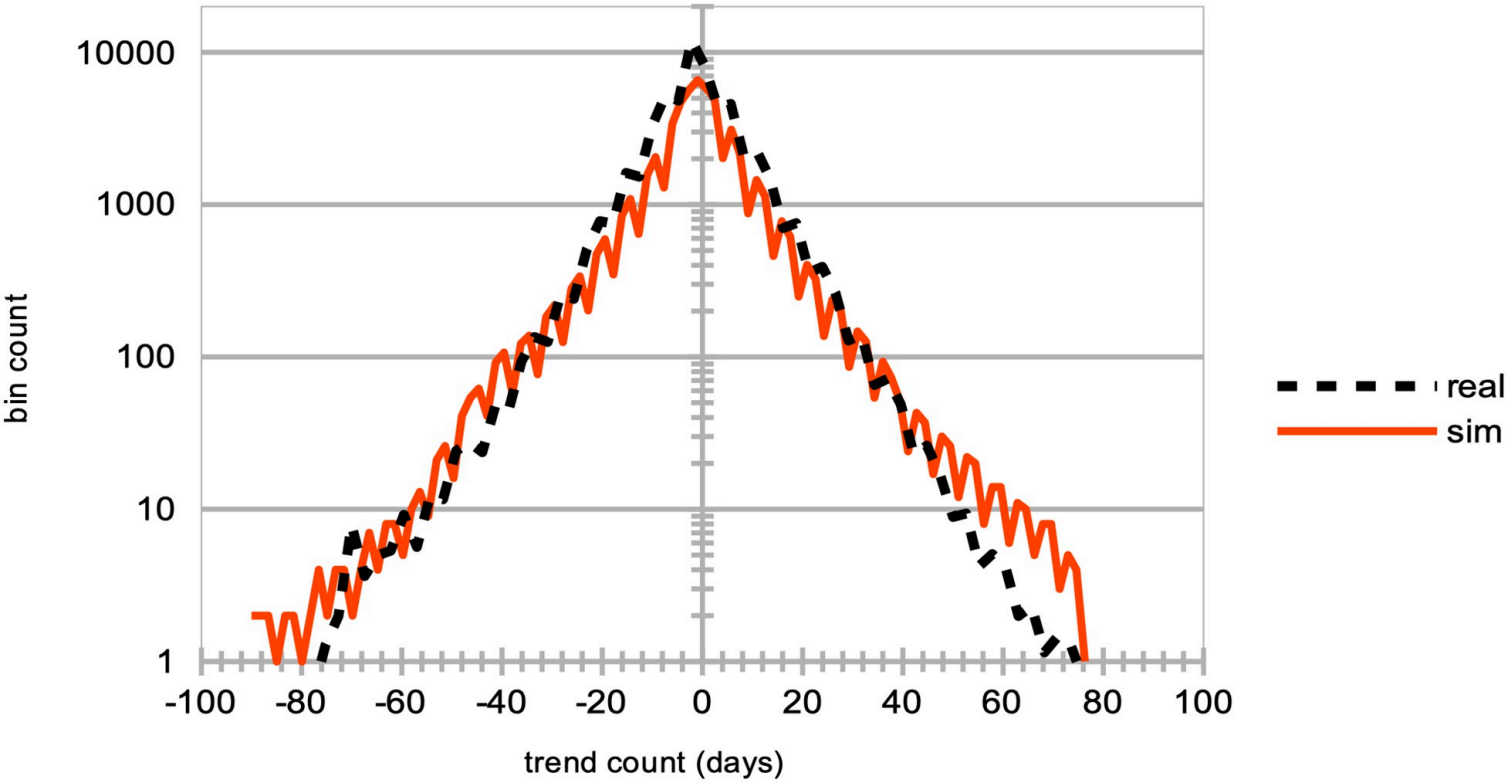
Distribution of consecutive days of price trends. This figure depicts the distribution of the number of consecutive days of increasing prices (positive values) and decreasing prices (negative values). The data is presented for both real (dashed black curve) and simulated (continuous red curve) scenarios. The simulations were generated using parameters *I* = 500, *J* = 1, *T* = 2875, and *S* = 20.

Here we present a list of crucial market microstructure indicators related to the calibration of the MAS simulator on Table 3. Figs 14 to 20 showcase the qualitative agreement in shape between the curves generated by our simulator and those derived from actual stock market data. When compared to stock market emulation [90, 91], our model underlines the effectiveness of reinforcement learning as a framework for describing agent learning and trading processes in stock markets. Unless explicitly mentioned, the following results are obtained from simulations conducted with *I* = 500 agents, *J* = 1 traded stock, *T* = 2875 time steps per simulation (equivalent to approximately a decade of trading days), and a total of *S* = 20 simulation runs.

- Fig 14 and Table 4 give the distribution of logarithmic returns of prices log[*P*(*t*)/*P*(*t* − 1)] for real (dashed black curve) and simulated (continuous red curve) data. It is evident that there is a close match between the simulated and real logarithmic price returns. However, one should note the limited variability of extreme events in the tails of the distribution, as revealed by the logarithmic y-axis.
- In Fig 15 and Tables 5–7, we examine the distributions of price volatilities over different time intervals: two weeks (black), three months (red), and one year (blue), for both real (dashed curves) and simulated (continuous curves) data. These volatilities are computed as standard deviations of prices normalized by the price itself, *σ*/*P*(*t*). We observe that emulating real volatilities at longer time scales is more challenging, likely due to our real data sample covering a unique and exceptional market period during the years 2008–2009, namely the Global Financial Crisis.
- Fig 16a and Tables 8–10 illustrate the distributions of correlations in the price logarithmic returns between distinct intervals [*t* − Δ, *t*] and [*t* − 2Δ, *t* − Δ] at each time step *t*, considering values of Δ corresponding to two weeks (black), three months (red), and one year (blue). This analysis is conducted for both real (dashed curves) and simulated (continuous curves) data. Despite the overall good fit, especially concerning the general shape of the distributions, the presence of numerous zero autocorrelations in real data raises questions. We suggest that this could be attributed to specific intraday market activity or the absence of trading volumes over extended periods for stocks of smaller capitalization companies.
- Similarly, Fig 17 and Table 11 show the simulated data (continuous curves) emulating real data (dashed curves) in terms of the asymmetric shape of the distribution of price volatility correlations between separated intervals [*t* − Δ, *t*] and [*t* − 2Δ, *t* − Δ] for Δ = 2*T*_*w*_ at each time step *t*. Additionally, Fig 16b showcases the resemblances in the shapes of the distributions of trading volume correlations between these same separated intervals, considering values of Δ corresponding to two weeks (black), three months (red), and one year (blue), albeit with some differences in zero values.
- Fig 18 presents the distributions of correlations in price logarithmic returns at each time step *t* for simulated data (continuous curves) and real data (dashed curves) between blended intervals [*t* − *T*_*w*_, *t*] and [*t* − *T*_*w*_ − *δ*, *t* − *δ*] for shifts *δ* of one day (black), two days (red), three days (blue), four days (green), and five days (yellow). Again, some differences in zero values are noticeable.
- In Fig 19a, we examine the means of blended correlations of logarithmic returns of prices at each time step *t* between intervals [*t* − *T*_*w*_, *t*] and [*t* − *T*_*w*_ − *δ*, *t* − *δ*] for shifts *δ* = 1, 2, 3, 4, 5, comparing the real (blue) and simulated (red) data. Similarly, Fig 19b demonstrates close fits for larger intervals of 2*T*_*w*_ instead of *T*_*w*_. These statistics are vital in understanding that the MAS generates a price microstructure that eliminates arbitrage opportunities and exhibits market memory through agent learning. In other words, agents learn to exploit short-term causal structures in historical prices.
- Fig 20 and Table 12 display the distribution of the number of consecutive days with increasing (positive values) and decreasing (negative values) prices at each time step *t*, for both simulated (continuous curve) and real (dashed curve) data. The count of consecutive days with increasing or decreasing prices serves as an insightful indicator of market sentiment, signaling whether the market is “bearish” or “bullish.” Apart from a few extreme bullish events, our MAS simulation effectively replicates the general dynamics of stock market prices observed in real data.

**Table 3:** Sum up of stylised facts in literature.

Stylised fact	Empirical studies
Non-gaussian price returns	[64, 92–95]
Volatility/volume clustering	[94, 96–100]
Price returns autocorrelation decay	[93, 94, 101–103]

**Table 4:** Statistics of the logarithmic price returns of Fig 14.

Moment	real	simulated
mean	-0.0048	-0.0117
standard deviation	0.0050	0.0347
skewness	-0.7505	0.0932
excess kurtosis	-1.3108	21.4473

**Table 5:** Statistics of the 2*T*_*w*_ price volatilities of Fig 15.

Moment	real	simulated
mean	0.0372	0.0271
standard deviation	0.0334	0.0215
skewness	-24.6570	10.0955
excess kurtosis	474.9102	86.7021

**Table 6:** Statistics of the 3*T*_*m*_ price volatilities of Fig 15.

Moment	real	simulated
mean	0.0654	0.0632
standard deviation	0.0621	0.0429
skewness	13.4716	3.8499
excess kurtosis	152.3164	17.5890

**Table 7:** Statistics of the *T*_*y*_ price volatilities of Fig 15.

Moment	real	simulated
mean	0.1311	0.1349
standard deviation	0.1171	0.0792
skewness	7.9590	2.7938
excess kurtosis	58.1109	10.3387

**Table 8:** Statistics of 2*T*_*w*_ autocorrelations of logarithmic price returns of Fig 16a.

Moment	real	simulated
mean	-0.0190	-0.0085
standard deviation	0.3101	0.3158
skewness	0.0108	0.0011
excess kurtosis	-0.2620	-0.3646

**Table 9:** Statistics of 3*T*_*m*_ autocorrelations of logarithmic price returns of Fig 16a.

Moment	real	simulated
mean	-0.0160	-0.0170
standard deviation	0.1225	0.1314
skewness	0.0384	0.0127
excess kurtosis	0.4026	-0.0588

**Table 10:** Statistics of *T*_*y*_ autocorrelations of logarithmic price returns of Fig 16a.

Moment	real	simulated
mean	-0.0116	-0.0061
standard deviation	0.0610	0.0600
skewness	-0.0194	-0.1751
excess kurtosis	0.5164	0.2897

**Table 11:** Statistics of 2*T*_*w*_ autocorrelations of price volatilities of Fig 17.

Moment	real	simulated
mean	-0.2088	-0.1981
standard deviation	0.5743	0.5538
skewness	0.4280	0.3943
excess kurtosis	-1.1023	-1.0964

**Table 12:** Statistics of the consecutive days of price trends of Fig 20.

Moment	real	simulated
mean	-1.1909	-0.8787
standard deviation	10.2580	11.9272
skewness	-0.000099	-0.0889
excess kurtosis	3.8487	5.5386

In summary, the calibration procedure demonstrates that SYMBA reproduces the distribution of logarithmic price returns, as depicted in Fig 14, and the autocorrelations at various time scales, illustrated in Figs 16a, 18, 19a and 19b. These autocorrelation measures are crucial in the calibration process, as they relate to the absence of arbitrage opportunities and market memory, which are fundamental characteristics of financial markets. In other words, beyond the stylized facts, the simulated data should not exhibit price patterns that are more easily discernible and exploitable for trading than those observed in real data, if any. Furthermore, we can emphasize that the MAS simulator faithfully replicates real stock market dynamics, including periods of recession and growth, as depicted in Fig 20. With that said, several avenues can be thought of for enhancing the model’s performance and properties:

- The extreme tail distribution of long-term price volatilities in Fig 15: These are the most challenging microstructure effects to capture, as they are related to jump diffusion processes inherent to volatile events in the life of a company, industry sector, or the overall market (it’s worth noting that the LSE data includes the financial crisis of 2008–2009).
- The peak in zero autocorrelations for real price returns and volatility in Figs 16a and 17: We attribute this phenomenon to the fact that the simulator does not account for intraday market activity, or it may be attributed to thinly traded small-cap companies.
- Heavier tails in the distributions of autocorrelation of trading volumes in Fig 16b: This is likely a result of seasonal and calendar effects specific to real stock markets.

## Supporting information

S1 Data (ZIP)
