# Correction: Mesoscale effects of trader learning behaviors in financial markets: A multi-agent reinforcement learning study

**DOI:** 10.1371/journal.pone.0319528

**Published:** 2025-02-12

**Authors:** Johann Lussange, Stefano Vrizzi, Stefano Palminteri, Boris Gutkin

There are errors in the Funding statemet and Acknowledgment section. The correct Funding statement and Acknowledgment section is as follows: We graciously acknowledge the support of ANR 1152 CogFinAIgent for this work. Parts of this research are also supported by the European Union’s 1153 Horizon 2020 research and innovation programme under the Marie Skłodowska-Curie 1154 grant agreement No 945304—Cofund AI4theSciences, hosted by PSL University. This study also benefits from funding by a grant from the RF Ministry of Science and Higher Education (grant ID: 075-15-2022-325) to HSE University.
